# Out-of-frame *CBX3::ALK* fusion drives ALK activation and therapy response

**DOI:** 10.1016/j.xcrm.2026.102697

**Published:** 2026-03-25

**Authors:** Jen-Fan Hang, Han-Ying Cheng, Yu-Shuen Tsai, Sin-Ying Lin, Jie-Hong Song, Chih-Hung Chung, Muh-Hwa Yang

**Affiliations:** 1 Department of Pathology and Laboratory Medicine, Taipei Veterans General Hospital, Taipei 112201, Taiwan; 2 Department of Pathology, School of Medicine, National Yang Ming Chiao Tung University, Taipei 112304, Taiwan; 3 Institute of Clinical Medicine, National Yang Ming Chiao Tung University, Taipei 112304, Taiwan; 4 Cancer and Immunology Research Center, National Yang Ming Chiao Tung University, Taipei 112304, Taiwan; 5 Department of Oncology, Taipei Veterans General Hospital, Taipei 112201, Taiwan

**Keywords:** melanoma, *CBX3*, *ALK*, out-of-frame, gene fusion, alectinib, alternative translation

## Abstract

Kinase gene fusions are critical oncogenic drivers and key targets in precision oncology. Here, we report a *CBX3::ALK* out-of-frame fusion identified in a case of metastatic melanoma, which produces functional ALK isoforms via alternative translation start sites. The patient demonstrates a remarkable clinical response to the ALK inhibitor alectinib. Functional studies confirm that the *CBX3::ALK*-derived isoforms retain oncogenic signaling and tumorigenic potential. Our findings reveal limitations in current tertiary analysis strategies for functional gene fusion detection that may overlook clinically relevant out-of-frame fusions. Additional analysis of pan-cancer RNA sequencing data from 5,725 tumors and genomic datasets comprising 6,977 melanomas demonstrates that such events are rare but potentially significant. This study highlights the need to consider alternative translation mechanisms and incorporate additional analytic filters, such as 5′/3′ expression imbalance, to better capture actionable out-of-frame fusion events in the era of precision oncology.

## Highlights

• Out-of-frame *CBX3::ALK* fusion generates functional ALK via alternative translation
• *CBX3::ALK* induces oncogenic signaling, cellular transformation, and tumor growth
• ALK inhibitors suppress *CBX3::ALK*-driven signaling and cell growth
• Rare out-of-frame kinase fusions can be clinically actionable in precision oncology

## Graphical abstract

**Figure:**
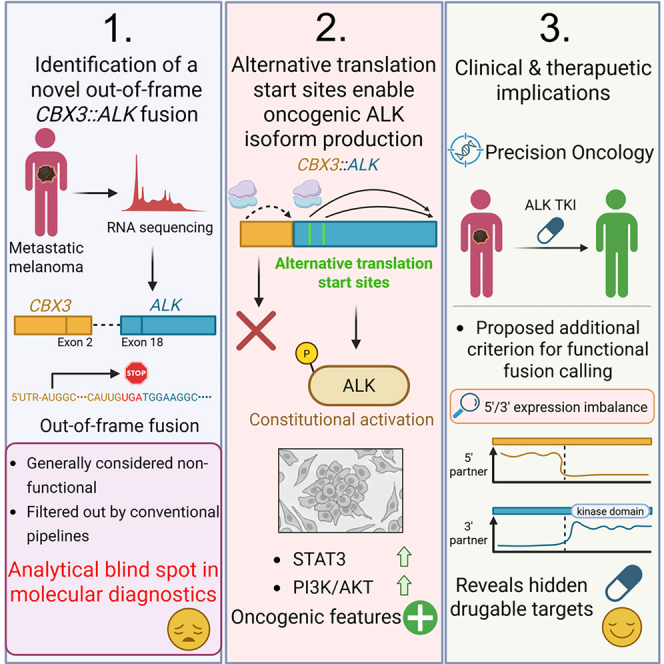

## Teaser

Hang et al. identify an out-of-frame *CBX3::ALK* fusion in metastatic melanoma that generates functional ALK isoforms through alternative translation start sites. This study demonstrates that rare noncanonical out-of-frame fusions can be oncogenic and therapeutically actionable, highlighting an analytic blind spot in current gene fusion detection pipelines.

## Introduction

Kinase gene fusions have emerged as critical genomic alterations in various cancers, offering significant implications for precision medicine.^1^ These fusions result from chromosomal rearrangements, leading to the formation of chimeric proteins with aberrant kinase activity that disrupt normal cellular signaling pathways, driving oncogenesis and providing potential therapeutic targets.^2^ Accurate identification and characterization of kinase gene fusions play a crucial role in precision medicine, enabling targeted therapies and guiding treatment decisions. Tertiary analysis frameworks apply bioinformatics-based fusion interpretation criteria to prioritize functional kinase gene fusions from high-throughput sequencing data, typically encompassing features such as strand orientation (in-strand), open reading frame (in-frame), and preservation of the functional kinase domain.^3^^,^^4^ Gene fusions that do not meet these criteria may be interpreted as nonfunctional and thus potentially ineligible for targeted therapies. Theoretically, out-of-frame fusions cause shifting of the open reading frame, often leading to premature stop codons, resulting in protein truncation or altered amino acid sequences.^5^^,^^6^ As a result, out-of-frame fusions are generally presumed nonfunctional and unlikely to yield active kinase proteins. This assumption has led to the common practice of filtering out such fusions in many clinically relevant fusion-calling pipelines, potentially precluding rare but clinically significant fusion events. In this study, we present a patient with metastatic melanoma harboring an out-of-frame *CBX3::ALK* fusion who exhibited an unusual and remarkable response to the ALK inhibitor alectinib. Our investigation further reveals that this out-of-frame fusion drives high-level translation of functional ALK isoforms through alternative translation start sites, uncovering an alternative mechanism of ALK kinase activation in solid tumors.

## Results

### Identification and characterization of CBX3:ALK out-of-frame fusion

**Figure 1:**
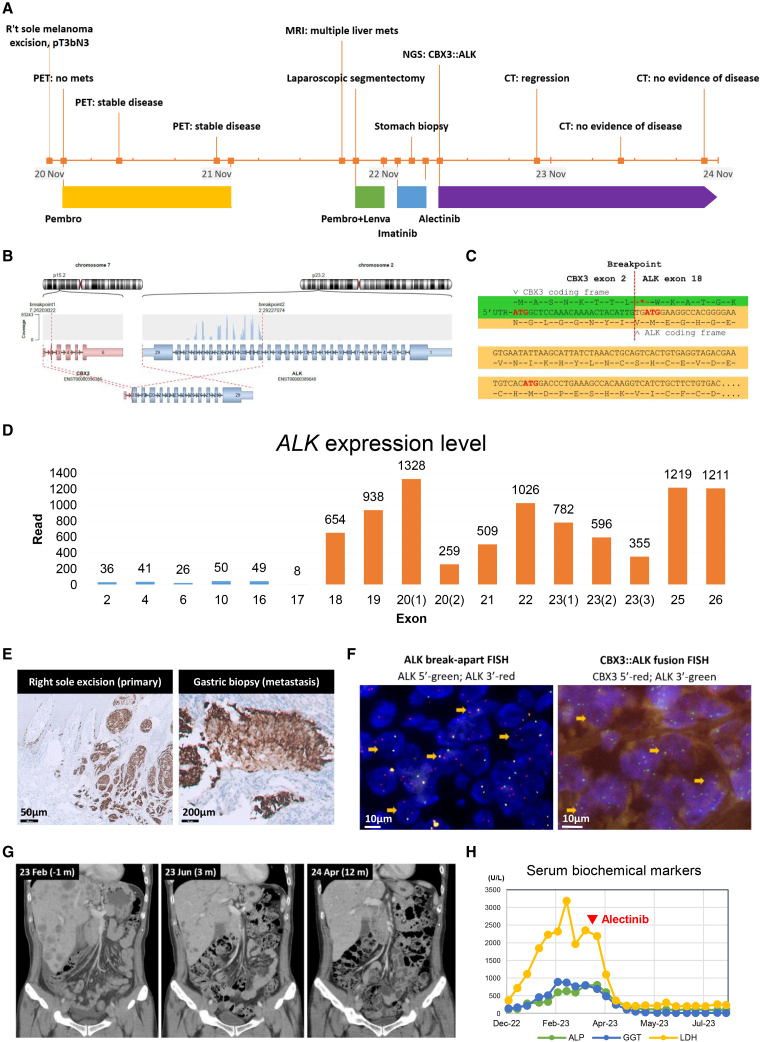
Identification of *CBX3::ALK* fusion gene in a metastatic melanoma patient with clinical response to alectinib (A) The clinical course and treatment of the patient. (B) Chromosomal rearrangement of the *CBX3::ALK* fusion. The exon 2 of *CBX3* was fused to exon 18 of *ALK*. (C) Schematic presentation of the breakpoint nucleotide sequence of the *CBX3::ALK* fusion. (D) Targeted RNA sequencing covering different regions of *ALK* demonstrated a drastic increase in absolute read counts beyond exon 18 (highlighted in orange bars). (E) Immunohistochemical staining of ALK in primary (left) and metastatic (right) melanoma. (F) Fluorescence *in situ* hybridization (FISH) for detection of the *CBX3::ALK* fusion in patient samples. Left: FISH using *ALK* break-apart probes. *ALK-*5′ probe: green; *ALK*-3′ probe: red. The yellow arrows indicate the break-apart *ALK*. Right: *CBX3::ALK* fusion probes. *CBX3*-5′ probe: red; *ALK*-3′ probe: green. The yellow arrows indicate the fusion. (G) Computed tomography scan images of the metastatic melanoma patient before (left), 3 months after (middle), and 12 months after (right) alectinib treatment. (H) Changes in serum levels of biochemical markers during treatment.

A 50 year-old Taiwanese male patient was diagnosed with stage IIIC malignant melanoma on the right sole in November 2020 and underwent surgical treatment. Initial genetic testing by Sanger sequencing revealed no *BRAF* mutation, but a *KIT* p.S451Y variant of undetermined significance was detected. The patient received adjuvant pembrolizumab for 1 year. However, in August 2022, multiple distant metastases were discovered in the lung, liver, stomach, and bone. Subsequent treatment with pembrolizumab plus lenvatinib and imatinib proved ineffective. The clinical course of the patient is illustrated in Figure 1A. To guide further treatment, a combined DNA- and RNA-based next-generation sequencing (NGS) cancer panel (ACTOnco+, ACT Genomics, Taipei, Taiwan) was performed on the metastatic gastric biopsy specimen, revealing a low tumor mutation burden (<1/Mb) and no clinically significant gene mutations, including the absence *KIT* p.S451Y variant. Unexpectedly, a *CBX3*(exon2)*::ALK*(exon18) fusion was identified (Figure 1B). Bioinformatics analysis indicated an out-of-frame fusion that introduced a stop codon immediately downstream of the fusion site (Figure 1C). Consequently, this fusion was initially considered nonfunctional and was not included in the official NGS report. Nevertheless, the molecular tumor board recommended further investigation due to potential therapeutic implications. Subsequent examination of RNA expression across *ALK* gene regions revealed an unusual 5′/3′ expression imbalance pattern, with a sharp transition between exons 17 and 18 (Figure 1D), indicating disproportionately high expression of the 3′ kinase domain. This pattern is often considered a surrogate marker of a functional driver fusion.^7^^,^^8^ In support of this, ALK immunohistochemistry (clone D5F3, Ventana) performed on both primary cutaneous tumor and metastatic lesions from liver and gastric biopsies demonstrated diffuse and strong cytoplasmic expression with H score of 300 (Figure 1E), suggesting overexpression of ALK protein with preservation of the kinase domain epitope from the onset of disease. Fluorescence *in situ* hybridization confirmed the presence of *CBX3::ALK* fusion, with 92% positive nuclei using the *ALK* break-apart probe and 52% positive nuclei using the *CBX3::ALK* fusion probes (Figures 1F and S1). Based on these findings, the patient was treated with ALK inhibitor alectinib, showing an excellent and sustained response with marked tumor size reduction within 3 months and near-complete remission after 1 year of treatment (Figures 1G and 1H). We performed the tumor response assessment according to the Response Evaluation Criteria in Solid Tumors (RECIST) v.1.1, comparing the target lesions identified at baseline (February 2023) with those at the most recent evaluation (June 2025). The sum of the longest diameters of all target lesions decreased from 18.77 cm at baseline to 0 cm at the latest assessment, indicating a confirmed complete response to alectinib.

### Alternative translation start sites enable oncogenic ALK isoform production

**Figure 2:**
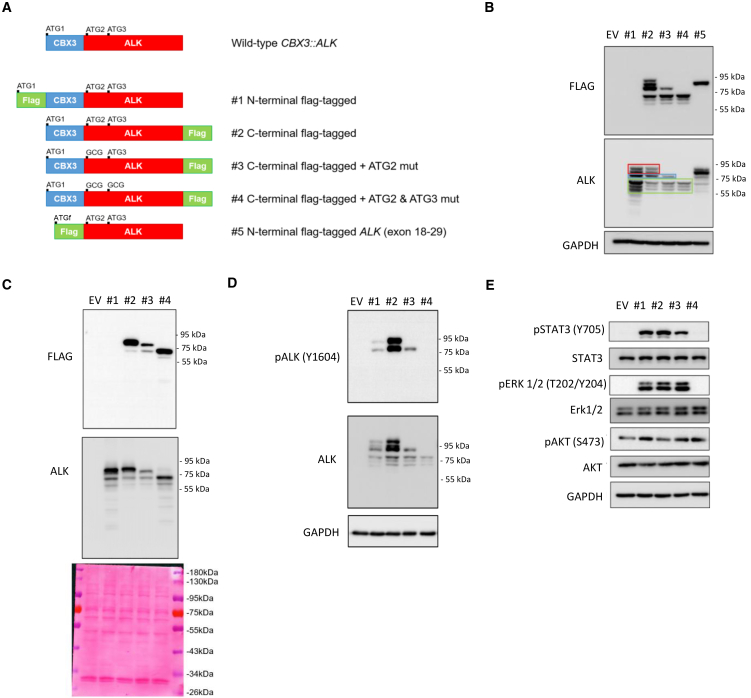
Alternative translation start sites enable oncogenic ALK isoform production in *CBX3::ALK* fusion (A) Schematic organization of the constructs of *CBX3::ALK* fusion for experiments. ATG1/ATG2/ATG3, the first, second and third ATG of *CBX3::ALK*; ATGf, artificial ATG for the N-terminal FLAG-tagged #5 construct. (B) Western blot analysis of FLAG-tagged and ALK protein in HEK293T cells transfected with the indicated constructs or empty vector control (EV). Red rectangle indicates the ALK isoforms using second ATG in exon 18 as the translation start site. Blue rectangle indicates the ALK isoforms using third ATG in exon 18 as the translation start site. Green rectangle indicates the ALK isoforms using other ATG in exon 18 as the start site. GAPDH is a protein loading control. (C) Detection of FLAG-tagged and ALK protein expression from an *in vitro* transcription/translation assay using immunoblotting. Ponceau S staining was a loading control (lower). (D) Western blots of ALK and phosphor-ALK (Y1604) of HEK293T cells transfected with the indicated constructs or empty vector control (EV). GAPDH is a protein loading control. (E) Western blots of the ALK-related signal molecules including total STAT3, phosphor-STAT3 (Y705), total ERK, phosphor-ERK1/2 (T202/Y204), total AKT, and phosphor-AKT (S473) in HEK293T cells transfected with the indicated constructs or empty vector control (EV). GAPDH is a protein loading control.

To elucidate the mechanism behind ALK kinase protein overexpression, we examined the fusion transcript sequence. We hypothesized that alternative translation initiation could occur at the second or subsequent ATG start sites within *ALK* exon 18 (Figure 1C). To investigate these potential translation start sites and functionally characterize the *CBX3::ALK* out-of-frame fusion, we generated HEK293T cell lines expressing the following constructs: (1) N-terminal FLAG-tagged *CBX3::ALK*, (2) C-terminal FLAG-tagged *CBX3::ALK*, (3) C-terminal FLAG-tagged *CBX3::ALK* with the second ATG (ATG2, equivalent to the 973 residues of the wild-type ALK protein) mutation, (4) C-terminal FLAG-tagged *CBX3::ALK* with ATG2 and the third ATG (ATG3, equivalent to the 997 residues of the wild-type ALK protein) mutations, and (5) N-terminal FLAG-tagged *ALK* (exon 18–29) (Figure 2A). Western blot analysis revealed comparable ALK expression levels in both N- and C-terminal FLAG-tagged *CBX3::ALK* constructs (#1 and #2), but the FLAG epitope was only detectable in cells expressing the C-terminal tag (Figure 2B), indicating that ALK kinase protein translation is not initiated from the first ATG (ATG1) within *CBX3*. Sequential mutagenesis of putative alternative translation start sites demonstrated that mutations at ATG2 (#3) and both ATG2 and ATG3 (#4) progressively reduced higher-molecular-weight translation products (Figure 2B). *In vitro* transcription/translation assays under cell-free conditions yielded similar blot patterns to those observed in HEK293T models, affirming the utilization of ATG2 and ATG3 as translation start sites, but not ATG1 (Figure 2C). Evaluation of ALK activation and downstream signaling showed that mutations at ATG2 (#3) and both ATG2 and ATG3 (#4) significantly impaired ALK phosphorylation capability at Tyr1064 compared to wild-type constructs (#1 and #2) (Figure 2D). Consequently, phosphorylation of downstream molecules STAT3 and ERK1/2 was notably disrupted, with the most pronounced effect observed in the *CBX3::ALK* construct harboring both ATG2 and ATG3 mutations (#4) (Figure 2E).

To characterize the subcellular distribution of the ALK isoforms generated by the *CBX3::ALK* fusion, we performed subcellular fractionation followed by western blot analysis. ALK protein was detected in the cytoplasmic and membrane fractions but was absent from the nuclear fraction (Figure S2). The presence of protein products with distinct molecular weights across these compartments suggests the expression of multiple ALK isoforms. Consistent with these findings, immunofluorescence analysis demonstrated cytoplasmic-predominant ALK localization (Figure S2). Collectively, these results indicate that the protein product of *CBX3::ALK* fusion does not retain the nuclear localization properties associated with *CBX3*.

### CBX3:ALK fusion demonstrates oncogenic potential *in vitro* and *in vivo*

**Figure 3:**
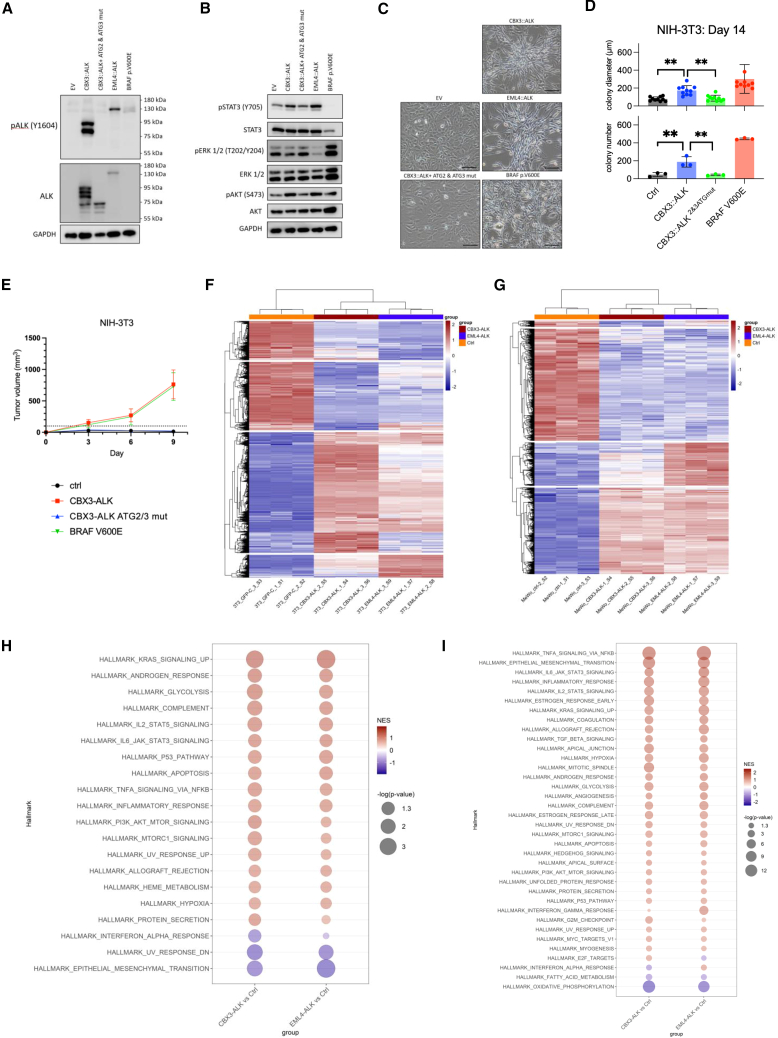
Characterization of the oncogenic potential of *CBX3::ALK* fusion (A) Western blots of total ALK and phosphor-ALK (Y1604) of NIH 3T3 cells transfected with an EV, plasmid encoding *CBX3::ALK* fusion, plasmid encoding *CBX3::ALK* fusion with mutation of second and third ATG sites, *EML4::ALK* fusion, and *BRAF* p.V600E. GAPDH is a protein loading control. (B) Western blots of ALK-related signal molecules including total STAT3, phosphor-STAT3 (Y705), total ERK, phosphor-ERK1/2 (T202/Y204), total AKT, and phosphor-AKT (S473) of NIH 3T3 cells transfected with an EV, plasmid encoding *CBX3::ALK* fusion, plasmid encoding *CBX3::ALK* fusion with mutation of second and third ATG sites, *EML4::ALK* fusion, and *BRAF* p.V600E. GAPDH is a protein loading control. (C) Phase contrast images showing the morphology of NIH 3T3 cells transfected with the indicated vectors (scale bar: 100 μm). (D) Soft agar colony formation assay for NIH 3T3 cells transfected with the indicated vectors at days 14 (Student’s *t* test, ∗∗*p* < 0.01). Data are presented as mean ± SD (diameter: *n* = 10; number: *n* = 3). (E) Tumor growth curves of xenografts derived from NIH 3T3 cells expressing the indicated constructs. Data are presented as mean ± SD (*n* = 3). (F and G) Hierarchical clustering heatmaps of significantly differentially expressed genes in NIH 3T3 (F) and MeWo (G) cells expressing control vector, *CBX3::ALK*, or *EML4::ALK*. *ALK* fusion-expressing cells clearly segregate from controls, with partially overlapping transcriptional profiles between *CBX3::ALK* and *EML4::ALK*. (H and I) GSEA of MSigDB Hallmark pathways for *CBX3::ALK* vs. control and *EML4::ALK* vs. control comparisons in NIH 3T3 (H) and MeWo (I) cells. Both *ALK* fusion models show significant enrichment of key oncogenic pathways, including KRAS-MAPK, STAT5, and PI3K-AKT-mTOR targets.

To assess the oncogenic potential of *CBX3::ALK*, we stably transduced NIH 3T3 cells with empty vector (EV), wild-type *CBX3::ALK*, or *CBX3::ALK* with ATG2 and ATG3 mutations. The canonical *EML4::ALK* fusion served as a control for constitutive ALK activation and oncogenic activity, while *BRAF* p.V600E was used as a control for oncogenic properties in melanoma. Western blot analysis revealed that the *CBX3::ALK* construct with ATG2 and ATG3 mutations lacked p-ALK expression and showed reduced downstream signaling, particularly p-STAT3, compared to wild-type *CBX3::ALK* and *EML4::ALK* (Figures 3A and 3B). To extend these findings to melanoma-relevant systems, we expressed wild-type *CBX3::ALK* in two human melanoma cell lines: CA11 (acral melanoma; *BRAF/NRAS* wild type)^9^ and MeWo (fibroblast-like metastatic melanoma; *BRAF/NRAS* wild type). In both models, *CBX3::ALK* expression led to activation of downstream signaling pathways (Figure S3).

Phenotypically, NIH 3T3 cells expressing wild-type *CBX3::ALK*, *EML4::ALK*, or *BRAF* p.V600E demonstrated pronounced morphologic transformation, including loss of contact inhibition and blunted cellular processes, whereas cells expressing EV or *CBX3::ALK* with ATG2 and ATG3 mutations retained a non-transformed appearance (Figure 3C). Consistent with these observations, soft agar colony formation assays demonstrated significantly higher colony numbers and larger colony diameters in cells expressing wild-type *CBX3::ALK* and *BRAF* p.V600E compared to those expressing *CBX3::ALK* with ATG2 and ATG3 mutations (Figures 3D and S4).

To validate these findings *in vivo*, we performed xenograft tumor formation assays by subcutaneously injecting transduced NIH 3T3 cells into nude mice. Tumor formation occurred in mice injected with cells expressing wild-type *CBX3::ALK* and *BRAF* p.V600E, while no tumors developed in mice injected with cells transduced with EV or *CBX3::ALK* with ATG2 and ATG3 mutations (Figure S4). Tumor growth monitoring revealed significant increases in tumor volume for cells expressing wild-type *CBX3::ALK* and *BRAF* p.V600E, whereas mice injected with cells transduced with EV or *CBX3::ALK* with ATG2 and ATG3 mutations showed no tumor development throughout the observation period (Figure 3E).

### Transcriptomic profiling of ALK fusion-driven gene expression

To characterize the transcriptional consequences of *CBX3::ALK* activation and compare them with a canonical *ALK* fusion, we performed RNA sequencing (RNA-seq) on NIH 3T3 and MeWo cells expressing control vector, *CBX3::ALK*, or *EML4::ALK*. Differential expression analysis revealed distinct gene expression signatures associated with *ALK* fusion expression in both cellular contexts (Table S1). Unsupervised hierarchical clustering demonstrated clear separation of *ALK* fusion-expressing cells from vector controls, with *CBX3::ALK* and *EML4::ALK* showing partially overlapping transcriptional profiles (Figures 3F and 3G). Principal-component analysis further confirmed this segregation, indicating that *ALK* fusion status is a major driver of global transcriptomic variation (Figure S5). Gene set enrichment analysis (GSEA) identified significant enrichment of hallmark pathways commonly associated with oncogenic *ALK* signaling, including KRAS-MAPK, STAT5, and PI3K-AKT-mTOR targets, in both *CBX3::ALK*- and *EML4::ALK*-expressing cells (Figures 3H and 3I; Table S2). A Venn diagram of up-regulated genes highlighted substantial overlap between the two *ALK*-driven expression models, supporting the functional similarity between the noncanonical out-of-frame *CBX3::ALK* fusion and canonical in-frame *EML4::ALK* fusions (Figure S5).

### ALK inhibitors suppress CBX3:ALK fusion-induced signaling and cell growth

To evaluate the efficacy of ALK inhibitors in targeting the *CBX3::ALK* fusion, we treated NIH 3T3cells expressing this fusion with four ALK inhibitors: alectinib, brigatinib, ceritinib, and crizotinib, at various concentrations. Western blot analysis revealed a dose-dependent reduction in p-ALK and p-STAT3 expression across all treatment groups, indicating effective suppression of ALK-mediated signaling (Figures S6A and S6B). Cell viability assays demonstrated a significant decrease in cell survival with increasing concentrations of each ALK inhibitor, further confirming their efficacy in targeting ALK-driven oncogenic activity (Figure S6C). Additionally, soft agar colony formation assay showed a marked reduction in colony formation in cells treated with alectinib and brigatinib compared to the control, highlighting the potent anti-tumorigenic effects of these inhibitors (Figures S6D and S6E). These results collectively demonstrate that ALK inhibitors effectively suppress both ALK signaling and *CBX3::ALK* fusion-induced cell growth, underscoring their potential therapeutic value in targeting this fusion.

### Prevalence and significance of out-of-frame kinase gene fusions

**Figure 4:**
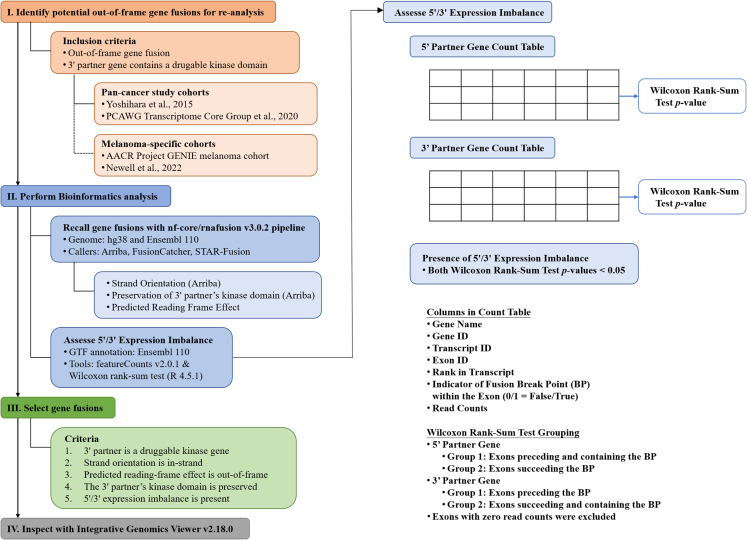
Analytical pipeline for identifying potentially functional out-of-frame fusions from publicly available RNA sequencing datasets

To investigate the incidence of out-of-frame kinase gene fusions as potential cancer drivers, we analyzed multiple genomic datasets. First, we examined the fusion database from our commercial NGS provider (ACT Genomics Co. Ltd), identifying 189 solid tumors with *ALK* fusions between 2021 and 2023. Among these, 10 cases had unreported fusion partners, with only one case (the current study) classified as out-of-frame (0.53%, 1/189). We then conducted a comprehensive literature review of The Cancer Genome Atlas (TCGA) pan-cancer RNA-seq datasets, focusing on two major study cohorts from Yoshihara et al. and the Pan-cancer Analysis of Whole Genomes Consortium, encompassing 5,725 cases.^10^^,^^11^ Using fusion-calling information from the supplementary data of these two studies, we focused on out-of-frame gene fusions in which druggable kinase genes serve as 3′ partner gene. As a result, 9 gene fusions were identified for further validations (first 9 fusion genes listed in Table S3), and the corresponding RNA-seq data were downloaded from the Cancer Genomics Hub (RRID: SCR_002657) for reanalysis. Three fusion detection tools, including Arriba,^4^ FusionCatcher, and STAR-Fusion,^12^ were used for validation. The workflow is illustrated in Figure 4 and described as follows. We first assessed whether these nine gene fusions could be detected using aforementioned analytic pipelines. With the exception of *C2orf61(STPG4)::ALK* (identified in sample TCGA-23-1027) that was not detected by STAR-Fusion, all fusions were consistently identified by all three tools. Of note, the supporting evidence for *C2orf61(STPG4)::ALK* was limited: FusionCatcher identified four spanning pairs and Arriba detected two split reads, indicating a low-confidence fusion event. We then confirmed that all gene fusions were oriented in the same transcriptional direction (in-strand). Subsequently, we evaluated the predicted reading frame for each fusion. Only two of the nine fusions (*MDM2::EGFR* and *UBE2Q2::NRG4*) were consistently predicted to be out of frame. Next, we analyzed the preservation of the kinase domain across all fusions. All retained the functional kinase domain, with the exception of *UBE2Q2::NRG4*. We further examined expression patterns associated with each fusion. As previously described, oncogenic fusions frequently result in disproportionate expression of retained exons, particularly in the 3′ partner gene, leading to a characteristic 5′/3′ read imbalance. The three analytic tools provide varying levels of annotation for fusion breakpoints: Arriba reports gene and transcript identifiers; FusionCatcher, gene and exon identifiers; and STAR-Fusion, gene identifiers alone. Because breakpoint coordinates vary slightly across tools, the exact fusion junction may differ among them. To evaluate 5′/3′ expression imbalance, we employed a Wilcoxon rank-sum test-based method and identified 5′/3′ imbalance in two fusions (*UACA::LTK* and *NFASC::NTRK1*, Figure S7; Table S4). These two fusions were predicted to be in-frame, which was further confirmed by manual review using the Integrative Genomics Viewer (Figure S7). However, these fusions have not been functionally characterized, and their biological significance and potential clinical relevance remain uncertain. In summary, we did not identify any analogous case in the public datasets of 5,725 pan-cancer cases that resembled our index case in terms of being a fusion initially predicted to be out of frame but exhibiting preserved 3′ kinase domain expression, suggestive of translation initiation at an alternative downstream start site. However, we reclassified seven cases, originally annotated as out of frame, to in frame. Among them, two cases demonstrated consistent 5′/3′ expression imbalance, further supporting their potential functionality. These findings highlight the utility of incorporating additional filters, such as 5′/3′ expression imbalance, into tertiary analysis strategies to better capture potentially actionable fusion events.

To further assess the frequency of this phenomenon in melanomas, we extended our analysis to two melanoma-specific datasets: the American Association for Cancer Research (AACR) Project GENIE melanoma cohort^13^ and the Australian melanoma cohort.^14^ In the GENIE cohort, only processed VCF/MAF files are publicly available, which precludes the reanalysis of raw FASTQ or aligned BAM data. Nevertheless, using the GENIE v.18.0 dataset accessed through cBioPortal, we observed that *ALK* structural variants (SVs) are exceedingly rare, occurring in only 0.2% (13 SVs among 6,407 melanoma cases). Although the available data do not allow assessment of *ALK* exon 18 3′-imbalanced fusions, this low frequency is consistent with the rarity of the fusion identified in melanomas.

In our analysis of the Australian melanoma cohort (*n* = 570), we examined the controlled whole-genome data from the International Cancer Genome Consortium. As reported by Newell et al., among 83,961 structural rearrangements detected through whole-genome sequencing (WGS), only one *ALK* fusion (*EML4::ALK*) was identified.^14^ However, RNA-seq data were not available, precluding assessment of 5′/3′ expression imbalance. To further evaluate the frequency of potentially functional out-of-frame fusions, we applied the same druggable gene-focused filtering strategy to this cohort. Of the 83,961 rearrangements, 32 met criteria for candidate loss-of-function events involving druggable 3′ partner genes, and 21 had matched RNA-seq data available. Reanalysis of these RNA-seq datasets identified only one rearrangement (*NRAF1::BRAF* of MELA_0296; see the last case in Table S3), showing concordance between RNA-seq and WGS fusion calls. However, this event did not fulfill all predefined selection criteria (Figure 4). Collectively, across these expanded melanoma datasets, we did not identify additional fusions analogous to *CBX3::ALK*, underscoring the extreme rarity of such events.

## Discussion

Our study presents a significant finding that challenges prevailing concepts in cancer genomics and precision medicine: an out-of-frame kinase gene fusion in solid tumors may produce a functional kinase protein and lead to a therapeutic response to selective kinase inhibitors. This discovery questions current assumptions in functional gene fusion detection and interpretation, with potential implications for the identifying actionable alternations and guiding patient care.

While other mechanisms of ALK activation in melanoma have been reported, our study is distinct in several key aspects. Beyond canonical in-frame *ALK* fusions, Wiesner et al. previously reported an ALK isoform (ALK^ATI^) in *ALK* fusion-negative melanomas, resulting from alternative transcription initiation in *ALK* intron 19.^15^ In contrast, our case involves an out-of-frame *CBX3::ALK* fusion that generates functional ALK isoforms through alternative translation start sites within the *ALK* coding sequence, rather than through alternative transcription initiation (Figure S8).

Our findings also related to two other reports. Cesi et al. identified a truncated ALK isoform in a laboratory-derived melanoma cell line (A375X1), which harbored a *BRAF* mutant and was resistant to BRAF inhibitors.^16^ In that model, ALK activation was driven by an out-of-frame gene fusion involving a 5′ sequence aligning with murine leukemia virus (MMLV) and a 3′ sequence starting from exon 18 of *ALK*. While this represents a functional out-of-frame *ALK* fusion, it was identified in an artificial, drug-resistant cell line and involved a viral sequence rather than a human gene, thereby limiting its clinical applicability. As such, it does not represent a true patient-derived case, and its relevance to human disease remains uncertain. Sadras et al. reported a case of B cell precursor acute lymphoblastic leukemia with an unusual *CD74::PDGFRB* out-of-frame fusion that generated a functional kinase protein through an alternative translation start site in *PDGFRB*.^17^ Although this case demonstrates the oncogenic potential of out-of-frame fusions through noncanonical mechanisms, it involves a hematopoietic malignancy, where gene fusions are frequently observed as primary drivers, rather than a solid tumor. In contrast, our study reports a clinically confirmed, targetable out-of-frame kinase fusion in a solid tumor, supported by functional validation and a documented therapeutic response.

Our study expands the landscape of actionable gene fusions beyond canonical in-frame events by demonstrating that certain out-of-frame fusions can produce oncogenic kinase proteins and elicit therapeutic responses. This challenges a foundational assumption in current tertiary analysis pipelines, which typically prioritize in-frame fusions and systematically filter out-of-frame events due to their presumed lack of biological or clinical relevance. However, our findings highlight that alternative translation mechanisms can rescue functional kinase activity from out-of-frame fusion transcripts, rendering these events oncogenic and, more importantly, therapeutically actionable.

To address this analytical blind spot, we propose refinements to current fusion-calling strategies. Incorporating additional analytic features, such as evaluating 5′/3′ expression imbalances, may improve the detection of noncanonical but clinically significant fusions. Furthermore, validation with complementary modalities, such as immunohistochemistry to assess protein overexpression, can provide accessible and practical confirmation of fusion-driven oncogenic signaling in clinical practice. Together, these strategies support the development of a broader and more nuanced framework for fusion analysis that ensures clinically actionable events are not overlooked.

In conclusion, we report an out-of-frame *CBX3::ALK* fusion in a patient with metastatic melanoma, which unexpectedly produce functional ALK isoforms through alternative translation start sites. Despite initial classification as nonfunctional, the fusion ultimately proved to be therapeutically actionable, as demonstrated by the patient’s remarkable response to the ALK inhibitor alectinib. This case exposes a critical limitation in current tertiary analysis pipelines for gene fusion detection, which tend to exclude out-of-frame fusions and may, therefore, miss clinically relevant events. To address this gap, fusion detection algorithms should be modified to account for alternative translation mechanisms in out-of-frame fusions. A more comprehensive understanding of fusion biology will improve the identification of rare but actionable fusions, ensuring that patients are not excluded from potentially life-saving targeted therapies.

### Limitations of the study

The primary limitation of this study is that it is based on a single index case, which restricts the generalizability of our findings. While the case provides compelling evidence that an out-of-frame *CBX3::ALK* fusion can produce a functional kinase protein and respond to targeted therapy, additional cases are needed to establish the broader clinical relevance of this mechanism. Despite our efforts to analyze a large dataset comprising 5,725 pan-cancer cases and 6,977 melanomas, we did not identify another example with the same fusion profile. This suggests that such events may be exceedingly rare or underreported due to limitations in current tertiary analysis pipelines. Nevertheless, our reanalysis led to the reclassification of seven cases initially annotated as out-of-frame fusions to in-frame configurations. Notably, two of these cases exhibited clear 5′/3′ expression imbalances, suggesting potential functional relevance. These observations underscore the need for more refined fusion-calling algorithms and highlight the challenges of detecting rare, noncanonical fusion events using current analytical frameworks. Future studies involving larger cohorts and orthogonal validation methods will be critical to further validate and expand upon our findings.

## Resource availability

### Lead contact

Further information and requests for resources and reagents should be directed to and will be fulfilled by the lead contact, Muh-Hwa Yang (mhyang2@nycu.edu.tw).

### Materials availability

The plasmids generated in this study are available from the lead contact upon reasonable request.

### Data and code availability

• The raw sequence data supporting the findings of this study are publicly available in the Sequence Read Archive (SRA; accession numbers: PRJNA1418094 and PRJNA1402121), which includes targeted DNA and RNA sequencing data from melanoma tumor tissue, and in the Gene Expression Omnibus (GEO: GSE311944), which contains transcriptome sequencing data from cell lines.
• This paper does not report the original code.
• Any additional information required to reanalyze the data reported in this work paper is available from lead contact upon request.

## Acknowledgments

The authors would like to express their gratitude to Dr. Ying-Hsia Chu (Department of Laboratory Medicine and Pathology, University of Minnesota Twin Cities, Minneapolis, Minnesota, USA) for the assistance with gene fusion classification, Dr. Che-Hung Shen (National Institute of Cancer Research, National Health Research Institutes, Tainan, Taiwan) for providing human melanoma cell lines CA11 and MeWo, Drs. Yi-Lin Hsieh and Hang-Che Yang (ACT Genomics Co., Ltd, Taipei, Taiwan) for providing melanoma tumor tissue-targeted RNA sequencing raw data and performing database search, and Dr. Yu-Chao Wang (Institute of Biomedical Informatics, National Yang Ming Chiao Tung University, Taipei, Taiwan) for the support in acquiring TCGA sequencing raw data. We thank National Core Facility for Biopharmaceuticals of the National Science and Technology Council, Taiwan, for providing sequencing services and technical support. This study utilized data generated by TCGA and the AACR Project GENIE. We thank the TCGA Research Network and the AACR GENIE Consortium for their commitment to data sharing. The interpretations and conclusions presented herein are solely the responsibility of the authors. The study was supported by the research grants from the 10.13039/100020595National Science and Technology Council, Taiwan (NSTC 114-2314-B-A49-068-MY3, 114-2320-B-A49-038, and 113-2634-F-039-001 to M.-H.Y. and 110-2320-B-075-003-MY3 and 114-2320-B-A49-033-MY3 to J.-F.H.); Cancer & Immunology Research Center, 10.13039/501100024990National Yang Ming Chiao Tung University (to M.-H.Y.) from The Featured Areas Research Center Program within the framework of the Higher Education Sprout Project by the Ministry of Education, Taiwan; and Taipei Veterans General Hospital, Taiwan (V113C-155 and V114C-065 to J.-F.H.).

## Author contributions

J.-F.H., conceptualization, resources, patient acquisition, methodology, investigation, writing – original draft, writing – review and editing; M.-H.Y., conceptualization, resources, patient acquisition, supervision, project administration, funding acquisition, writing – original draft, writing – review and editing; H.-Y.C., methodology, formal analysis, writing – original draft, writing – review and editing; S.-Y.L., methodology and investigation; Y.-S.T., methodology, investigation, formal analysis, data curation, writing – original draft, and writing – review and editing; J.-H.S., methodology, formal analysis, data curation, and writing – review and editing; C.-H.C., writing – original draft and writing – review and editing.

## Declaration of interests

The authors declare no competing interests.

## Declaration of generative AI and AI-assisted technologies in the writing process

During the preparation of this work, the authors used GPT-4o to assist with checking grammar and improving clarity. After using this tool, the authors carefully reviewed, edited, and approved all content and take full responsibility for the final version of this publication.

## STAR★Methods

### Key resources table

**Table:** 

REAGENT or RESOURCE	SOURCE	IDENTIFIER
**Antibodies**		

Rabbit monoclonal anti-GAPDH	Cell Signaling Technology	Cat # 2118; RRID: AB_561053
Rabbit polyclonal anti-STAT3	Cell Signaling Technology	Cat # 9139; RRID: AB_331757
Rabbit polyclonal anti-P-STAT3 (Tyr705)	Cell Signaling Technology	Cat # 9145; RRID: AB_2491009
Rabbit polyclonal anti-Erk1/2	Cell Signaling Technology	Cat # 9102; RRID: AB_330744
Rabbit polyclonal anti-P-Erk1/2 (Thr202/Tyr204)	Cell Signaling Technology	Cat # 9101; RRID: AB_331646
Rabbit polyclonal anti-AKT	Cell Signaling Technology	Cat # 9272; RRID: AB_329827
Rabbit polyclonal anti-P-ALT (S473)	Cell Signaling Technology	Cat # 9271; RRID: AB_329825
Rabbit polyclonal anti-ALK	Cell Signaling Technology	Cat # 3633; RRID: AB_11127207
Rabbit polyclonal anti-P-ALK (Tyr1604)	Cell Signaling Technology	Cat # 3341; RRID: AB_331047
Mouse monoclonal anti-FLAG	Sigma-Aldrich	Cat #F1804; RRID: AB_262044
Rabbit monoclonal anti-ALK (D5F5)	Roche Diagnostics	Cat # 066790720; RRID: AB_3675539
N-cadherin	BD Biosciences	Cat# 610920, RRID: AB_2077527
Histone H3	Cell Signaling Technology	Cat #9715S; RRID: AB_331563

**Bacterial and virus strains**		

ECOS™ St Competent Cells [HB101 Derived]	YB Biotech	XR-LYE307

**Biological samples**		

Human skin tumor tissue from adult patient with stage IIIC malignant melanoma used for targeted NGS cancer panel (*n* = 1)	Taipei Veterans General Hospital, Taipei, Taiwan	N/A

**Critical commercial assays**		

Ion AmpliSeq Library Kit	Thermo Fisher Scientific	Cat # 4480441
ArcherDX Lung FusionPlex Kit	ArcherDX	Cat # SK0133
OptiView DAB IHC Detection Kit	Roche Diagnostics	Cat # 06396500001
Vysis ALK Break Apart FISH Probe Kit	Abbott Molecular	Cat # 31-190068
TNT® Quick Coupled Transcription/Translation System	Promega	Cat #L1170
CCK-8 assay	Bioman	Cat # CK8001

**Deposited data**		

Targeted DNA and RNA sequencing data from melanoma tumor tissue	This paper	SRA: PRJNA1418094 and PRJNA1402121
Transcriptome sequencing data from cell lines	This paper	GEO: GSE311944
Human reference genome Ensembl release 110, GRCh38	Ensembl	https://ftp.ensembl.org/pub/release-110/fasta/homo_sapiens/dna/Homo_sapiens.GRCh38.dna_sm.primary_assembly.fa.gz
Ensembl Gene Transfer File release 110, GRCh38	Ensembl	https://ftp.ensembl.org/pub/release-110/gtf/homo_sapiens/Homo_sapiens.GRCh38.110.gtf.gz
COSMIC (version 86)	Forbes et al.	https://cancer.sanger.ac.uk/cosmic/download/cosmic
Genome Aggregation Database (version v2.0.2)	Karczewski et al.	https://gnomad.broadinstitute.org/downloads
TCGA RNA-seq bam files	Wilks et al.	https://gdc.cancer.gov/
RNA-seq bam files of the Australian melanoma cohort	Newell et al.	EGA: EGAD00001008837

**Experimental models: Cell lines**		

HEK293T	ATCC	CRL-3216; RRID: CVCL_0063
NIH3T3	ATCC	CRL-1658; RRID: CVCL_0594
CA11	Che-Hung Shen	Hsieh et al,^9^ 2023
MeWo	ATCC	HTB-65; RRID: CVCL_0445

**Experimental models: Organisms/strains**		

Mouse:CAnN.Cg-Foxn1^nu^/CrlNarl (nude mice), eight-week-old female mice were used in this study.	National Laboratory Animal Center, Taipei, Taiwan	N/A

**Oligonucleotides**		

Custom-designed CBX3:ALK fusion probe	Abnova, Taipei, Taiwan	N/A
Forward primer for CBX3:ALK 2ATG point mutation: CTACATTGTGGCGGAAGGCCACG	This paper	N/A
Reverse primer for CBX3:ALK 2ATG point mutation: CGTGGCCTTCCGCCACAATGTAG	This paper	N/A
Forward primer for CBX3:ALK 3ATG point mutation: GAATGTCACGCGGACCCTGA	This paper	N/A
Reverse primer for CBX3:ALK 3ATG point mutation: TCAGGGTCCGCGTGACATTC	This paper	N/A

**Recombinant DNA**		

*CBX3::ALK* fusion cDNA	Tri-I Biotech Inc, New Taipei City, Taiwan	N/A
*EML4::ALK* cDNA (pLenti-EML4-ALK variant 1)	Addgene	#183828; RRID: Addgene_183828
pCDH-CMV-MCS-EF1α-Puro	System Biosciences	Cat # CD510B-1
pCDH-MSCV-MCS-EF1α-GFP	System Biosciences	Cat # CD511B-1
pTnT™ Vector	Promega	Cat #L5610

**Software and algorithms**		

Nextflow (version 24.04.4)	Di Tommaso et al.	https://www.nextflow.io/; RRID: SCR_024135
nf-core rnafusion (version 3.0.2)	nf-core	https://nf-co.re/rnafusion/3.0.2/
Arriba (version 2.4.0)	Uhrig et al.	https://github.com/suhrig/arriba; RRID: SCR_025854
FusionCatcher (version 1.33)	Nicorici et al.	https://github.com/ndaniel/fusioncatcher; RRID: SCR_000060
STAR-Fusion (version 1.12.0)	Haas et al.	https://github.com/STAR-Fusion/STAR-Fusion; RRID: SCR_025853
SAMTOOLS (version 1.5)	GitHub	https://github.com/samtools/samtools; RRID: SCR_002105
featureCounts (version 2.0.1)	Liao et al.	https://subread.sourceforge.net/; RRID: SCR_012919
Integrative Genomics Viewer (version 2.18.0)	Robinson et al.	https://igv.org/; RRID: SCR_011793
GraphPad Prism (version 9)	GraphPad Software	https://www.graphpad.com/; RRID: SCR_002798

### Experimental model and subject participant details

#### Human patient

The patient was a 50 year-old Taiwanese male at the initial disease presentation. Formalin-fixed paraffin-embedded metastatic tumor tissue from the stomach were retrieved from the Pathology Archives of Taipei Veterans General Hospital. Written informed consent was obtained, with experiments performed under the approval of the Institutional Review Board (IRB) of Taipei Veterans General Hospital (IRB No.: 2024-06-018AC).

#### Mice

CAnN.Cg-Foxn1nu/CrlNarl mice were obtained from the National Laboratory Animal Center, Taipei, Taiwan. All animal experiments adhered to the guidelines and protocols approved by the Institutional Animal Care and Utilization Committee of the National Yang Ming Chiao Tung University (IACUC certificate No. 1110416). Female mice aged 8 weeks were used in all experiments.

#### Cell lines

Human embryonic kidney 293T cells (RRID: CVCL_0063), mouse embryonic fibroblast NIH3T3cells (RRID: CVCL_0594), human melanoma cell line MeWo (RRID: CVCL_0445) and human melanoma cell line CA11 (a kind gift from Dr. Che-Hung Shen)^9^ were used in this study. 293T and NIH3T3cells were cultured in Dulbecco’s Modified Eagle Medium (DMEM), while MeWo and CA11 cells were maintained in RPMI-1640 medium. All culture media were supplemented with 10% fetal bovine serum (FBS) and 1% penicillin-streptomycin. Cells were maintained at 37°C in a humidified atmosphere containing 5% CO2. The cells used in the experiments were tested negative for mycoplasma contamination and were authenticated via STR profiling.

### Method details

#### Targeted next-generation sequencing (NGS) cancer panel

Targeted DNA and RNA NGS was performed at a College of American Pathologist-accredited laboratory (ACT Genomics, Taipei, Taiwan) using the ACTOnco+ panel, which covers 440 cancer-related genes, as previously described.^18^ In brief, genomic DNA (gDNA) and total RNA were extracted from formalin-fixed, paraffin-embedded (FFPE) tumor samples. Forty nanograms of gDNA were amplified using multiplex PCR with the Ion AmpliSeq Library Kit (Thermo Fisher Scientific, Waltham, MA). Libraries were prepared, barcoded, and sequenced on an Ion Proton or Ion S5 sequencer using the Ion PI chip. Sequence reads were aligned to the human reference genome (hg19), and variant calling was performed using the Torrent Variant Caller plugin. Variants were annotated with the Variant Effect Predictor^19^ (VEP, version 88, RRID: SCR_007931) using COSMIC^20^ v.86 (RRID: SCR_002260) and Genome Aggregation Database^21^ r2.0.2 (RRID: SCR_014964) as references. Criteria for further variant analysis included at least 25 variant reads and an allele frequency of at least 2% for actionable variants and 5% for other variants. Polymorphisms were excluded using an in-house normal sample database. Copy-number variations (CNVs) were analyzed using ADTEx^22^ (RRID: SCR_012059), with amplification defined as copy number ≥4 and loss as ≤1. For RNA sequencing, extracted RNA was reverse-transcribed and subjected to library construction using the ArcherDX Lung FusionPlex kit (ArcherDX, Boulder, CO). Sequencing was performed according to Ion S5 sequencer protocol (RRID: SCR_026508). To ensure sequencing quality for fusion variant analysis, the average RNA Start Sites (SS) per control Gene Specific Primer 2 (GSP 2) should be ≥10. The fusion analysis pipeline aligned sequenced reads to the human reference genome, identified regions that map to noncontiguous regions of the genome, applied filters to exclude probable false-positive events and, annotated previously characterized fusion events according to Quiver Gene Fusion Database (ArcherDX). The targeted DNA and RNA sequencing data generated in this study have been deposited in the National Center for Biotechnology Information (NCBI) Sequence Read Archive (SRA) and are accessible under the accession numbers SRA: PRJNA1418094 and PRJNA1402121, respectively.

#### Immunohistochemistry

ALK protein expression was evaluated on FFPE tissue sections using the Ventana anti-ALK (D5F3) monoclonal primary antibody (RRID: AB_3675539) and the OptiView DAB Detection Kit on the Roche Ventana BenchMark ULTRA IHC/ISH System (RRID: SCR_025506). Staining was performed according to the manufacturer’s protocols. ALK immunoreactivity was semiquantitatively assessed using the H-score method.

#### Fluorescence *in situ* hybridization (FISH)

FISH was performed on FFPE tissue sections using the Vysis *ALK* break-apart probe (Abbott Molecular Inc., Des Plaines, IL) and a custom-designed *CBX3::ALK* fusion probe (Abnova, Taipei, Taiwan). The *ALK* break-apart probe detects chromosomal rearrangements involving the *ALK* locus, while the *CBX3::ALK* fusion probe specifically targets both partner genes. Tissue sections were deparaffinized, pretreated, and hybridized with probes following the manufacturers’ protocols. Post-hybridization washes were performed, and nuclei were counterstained with DAPI. Fluorescence signals were analyzed using a Zeiss Axio Imager 2 (RRID: SCR_018876) equipped with appropriate filters. Image capture and signal interpretation were performed using AxioVision Imaging System (RRID: SCR_002677). Positive results were confirmed by the presence of split or fusion signals in >20% of 50 non-overlapping nuclei of the tumor cells.

#### *CBX3:: ALK* Ffsion Ggne and Rrlated Ppasmid Ccnstruction

The *CBX3::ALK* fusion cDNA (Figure S9) was synthesized by Tri-I Biotech Inc (New Taipei City, Taiwan). The synthesized *CBX3::ALK* insert was cloned into either the pCDH-CMV-MCS-EF1α-Puro (System Biosciences, Palo Alto, CA) or the pCDH-MSCV-MCS-EF1α-GFP (System Biosciences, Palo Alto, CA) vectors (Figure S10). Multiple *CBX3::ALK* constructs were generated to examine fusion protein expression and function, including variations in epitope tag positioning and site-directed mutagenesis of alternative translation start sites, as detailed in Figure 2A. Primers used for site-directed mutagenesis are listed in the key resources table. For construction of the canonical *ALK* fusion control, *EML4::ALK* cDNA (variant 1) was obtained from pLenti-EML4-ALK (RRID: Addgene_183828) and subcloned into the pCDH-CMV-MCS-EF1α-GFP vector.

#### Western Blot

Cells were lysed in RIPA (50 mM Tris HCl, pH 7.4; 150 mM NaCl; 1 mM EDTA; 1% NP-40; 0.1% SDS; 0.5% sodium deoxycholate) buffer with 1X proteinase inhibitor from Roche (Mannheim, Germany) and incubated on ice for 20 min. Next, cell lysates were transferred into new Eppendorf tubes and vortexed for 1 min. After vertexing, the lysates were centrifuged at 12,500 rpm for 10 min, and the supernatants were collected. After centrifugation at 20,000 g for 10 min, the supernatants were collected. The quantity was determined with an infinite M200 (RRID: SCR_024560) using Pierce BCA protein assay (Thermo Fisher Scientific). All samples were diluted to equal protein concentrations by adding a proper volume of RIPA buffer. To break the protein structure, 6X sample buffer was added to each sample and mixed. The mixtures were heated at 95°C for 5 min. Denatured proteins were loaded in 6%–12% SDS–PAGE gels to separate the proteins with running buffer. A PVDF membrane from Millipore (Billerica, MA) was used to transfer protein samples from the gels to the membrane. The transfer system was used at 300 mA on ice for 2 h. Membranes containing denatured proteins were blocked in TBST with 5% skim milk at room temperature for 1 h. After blocking with milk, all membranes were washed 3 times for 10 min each with TBST. Then, all membranes were incubated with particular primary antibodies at 4°C overnight. The membrane was washed in TBST and incubated with secondary antibodies in 5% skim milk for 1 h at room temperature. The membrane was washed in TBST again and then incubated with ECL from Millipore (Billerica, MA). The results were measured using a GE LAS-4000 (GE Healthcare Inc., Marlborough, MA).

#### *In vitro* transcription and translation assay

For the cell-free *in vitro* transcription and translation assay, the TNT Quick Coupled Transcription/Translation System (Promega, Madison, WI) was used. Briefly, 500 ng of DNA template was added to each reaction, and the reactions were incubated at 30°C for 30 min. Denaturation was performed by adding 5X sample buffer, followed by incubation at 70°C for 10 min. Subsequently, 1/25 of the total sample was loaded onto a gel for western blot analysis. Ponceau S staining was used as a loading control. The input DNA template was designed using the pTnT Vector (Promega). ALK-related cDNA constructs were cloned into the pTnT Vector, while an empty pTnT Vector was used as a control.

#### Cell viability assay

Cell viability was assessed using the CCK-8 assay (Bioman, New Taipei City, Taiwan). Briefly, cell suspensions were seeded into 96-well plates in 100 μL of complete culture medium per well, with or without the test compounds. Following treatment, 10 μL of CCK-8 solution was added to each well, and the plates were incubated for an additional 1 h at 37°C. After incubation, measure the absorbance at 450 nm with a microplate reader. Cell viability was calculated as a percentage of the untreated control. All experiments were performed in triplicate and data were expressed as mean ± standard deviation (SD).

#### Soft agar colony formation assay

Anchorage-independent growth was assessed using a soft agar colony formation assay. A base layer was prepared by mixing Dulbecco’s Modified Eagle Medium (DMEM, Thermo Fisher Scientific) with 10% fetal bovine serum (FBS) and 0.5% agarose, which was plated into each well of a 6-well culture plate and allowed to solidify at room temperature. A top layer consisting of DMEM supplemented with 10% FBS, 0.3% agarose, and NIH3T3cells (10,000 cells per well) was then overlaid onto the base layer. The plates were incubated at 37°C in a humidified incubator with 5% CO_2_ for 14 days to allow for colony formation. Fresh growth medium was carefully added every 3–4 days to maintain optimal culture conditions. After the incubation period, colonies were stained with 0.005% crystal violet to enhance visualization and were then counted and measured using ImageJ software (RRID: SCR_003070). All experiments were performed in triplicate, and data were presented as mean ± standard deviation (SD).

#### Xenograft tumor forming

For tumor xenograft experiments, NIH3T3cells were transfected with PCDH-GFP-related plasmids, including control, *CBX3::ALK*-Flag, *CBX3::ALK* ATG2 and ATG3 mut-Flag, and *BRAF* p.V600E constructs. After expression, the cells were sorted using a CytoFLEX SRT cell sorter (RRID: SCR_025068). A total of 3 × 10^6^ NIH3T3cells were subcutaneously injected into 8-week-old female nude mice. Tumor volumes were measured every 3 days after injection and calculated using the formula: 0.5 × (length × width^2^).

#### RNA sequencing and data analysis

For RNA sample preparation: NIH3T3 and MeWo cells expressing a control vector, *CBX3::ALK*, or *EML4-ALK* were harvested for RNA extraction 48 h post-seeding, at which point cell confluence reached approximately 70–80%. Total RNA was extracted, and library preparation was performed by Illumina Stranded mRNA Prep kit. For sequencing and alignment, the cDNA libraries were sequenced on an Illumina NextSeq 2000 platform to generate single-end reads. Raw sequencing data processing and alignment were conducted using CLC Genomics Workbench (25.0.3; QIAGEN). Low-quality reads and adapter sequences were trimmed, and the remaining high-quality reads were mapped to the Mus musculus (GRCm39) or Homo sapiens (GRCh38) reference genomes for NIH3T3 and MeWo samples, respectively. Gene expression levels were quantified as raw read counts.

Differential expression analysis was performed using the DESeq2 package (version 1.42.1) in the R statistical environment. To identify significantly differentially expressed genes (DEGs), a cutoff of adjusted P-value <0.05 and | log2fc | >1 was applied. For downstream visualization, Principal Component Analysis (PCA) and hierarchical clustering heatmaps were generated using R. Additionally, Gene Set Enrichment Analysis (GSEA) was performed using the fgsea (version 1.28.0) to assess pathway enrichment patterns.

The RNA-sequencing data generated in this study have been deposited in the NCBI Gene Expression Omnibus (GEO) database and are accessible under the accession number GSE311944. The repository includes the raw sequencing flies, as well as the processed gene expression matrices generated by the CLC Genomics Workbench workflow.

#### Gene fusion bioinformatics analysis

To identify putative fusion genes, we used Nextflow v24.04.4 (RRID: SCR_024135)^23^ to run the nf-core^24^ rnafusion pipeline v3.0.2, which accepts FASTQ files as input and performs trimming, quality control, alignment, fusion gene detections with different tools, including Arriba v2.4.0 (RRID: SCR_025854),^4^ FusionCatcher v1.33 (RRID: SCR_000060), and STAR-Fusion v1.12.0 (RRID: SCR_025853),^12^ and visualization. Raw reads of the ACTOnco+ panel in bam format and aligned RNA-seq BAM files obtained from The Cancer Genome Atlas (RRID: SCR_003193) were converted to FASTQ files by SAMTOOLS v1.5 (RRID: SCR_002105). The human reference genome (hg38) FASTA file and the gene annotation GTF file were obtained from Ensembl release 110 (RRID: SCR_002344).^25^ All fusions of interest were rediscovered *de novo* by the pipeline. The predicted reading-frame status was reported directly by individual fusion-calling tools.

To investigate the 5′/3′ expression imbalance patterns of the putative fusion genes, we summarized the read counts of each exon with featureCounts v2.0.1 (RRID: SCR_012919).^26^ The exon order of corresponding transcripts, as annotated by Ensembl, was retrieved from BioMart. The analytical procedure is summarized as follows:1) Extract exon-level read counts for the reported transcript of each fusion partner; 2) Identify the breakpoint-containing exons; 3) Split exons into two groups (groups 1 and 2) based on preceding or succeeding the breakpoint. The breakpoint-containing exon belongs to group 1 or group 2, depending on whether it is the 5′ or the 3′ partner gene; 4) Perform a Wilcoxon rank-sum test comparing read counts between the groups (excluding zero-count exons); 5) Define *p* < 0.05 as indicating a significant imbalance; 6) Conclude that a fusion exhibits a 5'/3′ imbalance only when both partner genes meet significance criteria.

Selected gene fusions were inspected with Integrative Genomics Viewer v2.18.0 (RRID: SCR_011793).^27^ An ideal fusion of interest shall fulfill the five predefined criteria:1) 3′ partner is a druggable kinase gene; 2) Strand orientation is in-strand; 3) Predicted out-of-frame configuration; 4) Kinase domain of the 3′ partner is preserved; 5) Presence of 5'/3′ expression imbalance.

### Quantification and statistical analysis

#### Statistical anaylsis

All statistical analyses were performed using Student’s *t* test to compare differences between groups. A two-tailed *t* test was applied under the assumption of normal distribution. Results are reported as mean ± standard deviation (SD), unless otherwise specified. A *p*-value of <0.05 was considered statistically significant. Statistical significance levels are denoted as follows: *p* < 0.05 (∗), *p* < 0.01 (∗∗), *p* < 0.005 (∗∗∗). Data were graphed and analzed using GraphPad Prism 9 (RRID: SCR_002798).

## Supplemental information

Document S1. Figures S1–S10 and Tables S3 and S4

Table S1. Upregulated differentially expressed genes in cells expressing *CBX3::ALK* or *EML4::ALK*, related to Figure 3

Table S2. Hallmark list of GSEA analysis in NIH 3T3 and MeWo cells, related to Figure 3 (A) NIH 3T3 CBX3-ALK vs. control. (B) NIH 3T3 EML4-ALK vs. control. (C) MeWo CBX3-ALK vs. control. (D) MeWo EML4-ALK vs. control

Document S2. Article plus supplemental information
