# Supplementary material for: Out-of-frame CBX3::ALK fusion drives ALK activation and therapy response
Source: Cell Rep Med. 2026 Mar 25;7(4):102697. doi: 10.1016/j.xcrm.2026.102697 (PMC13130619; doi:10.1016/j.xcrm.2026.102697)
Supplement: Document S1. Figures S1–S10 and Tables S3 and S4 [file mmc1.pdf]

Cell Reports Medicine, Volume *7*

Supplemental information

Out-of-frame *CBX3::ALK* fusion drives ALK activation and therapy response

Jen-Fan Hang, Han-Ying Cheng, Yu-Shuen Tsai, Sin-Ying Lin, Jie-Hong Song, Chih-Hung Chung, and Muh-Hwa Yang

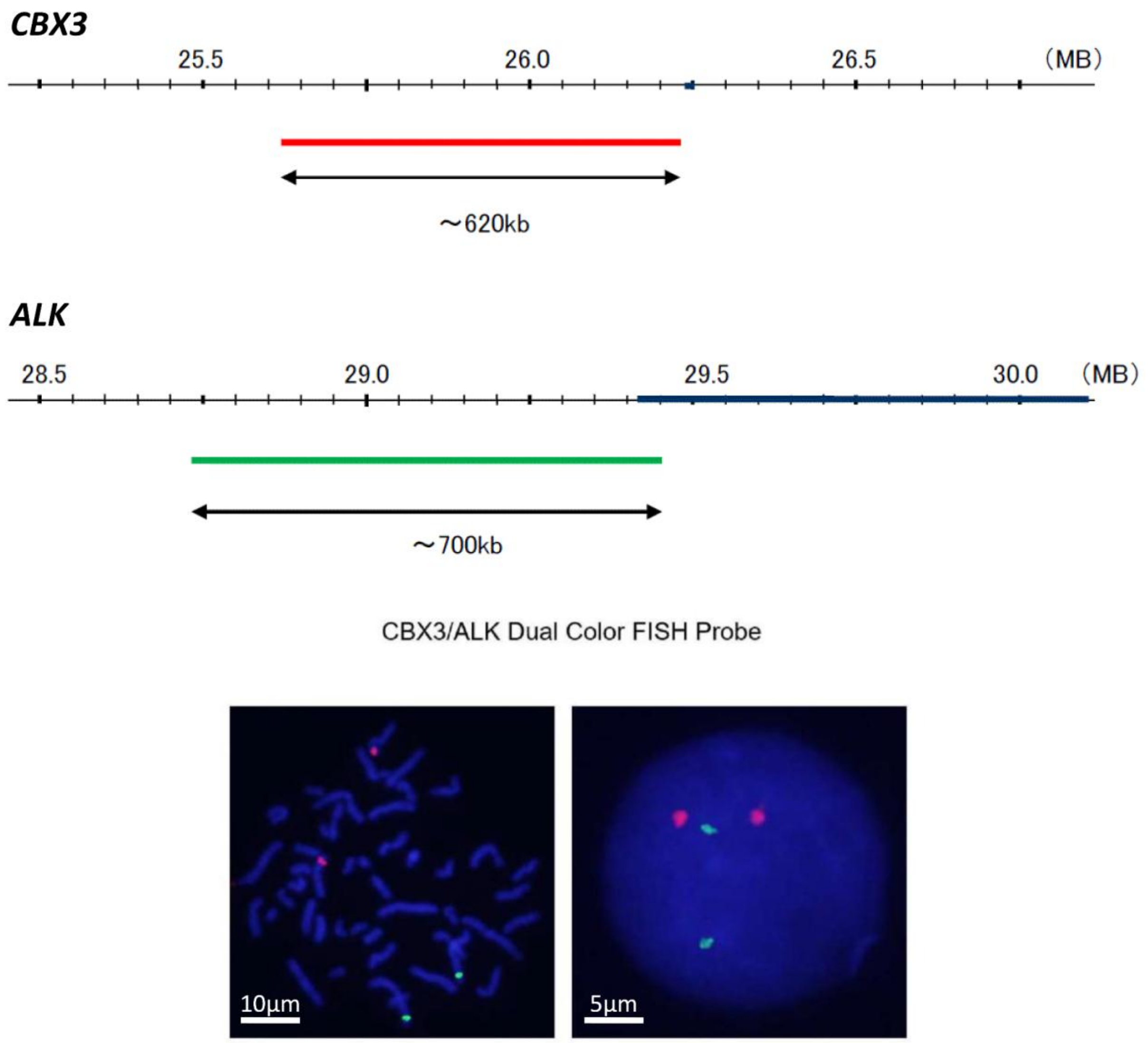

Supplementary Figure S1. Design and validation of the fluorescence in situ hybridization (FISH) probe for detection of *CBX3::ALK* fusion. Related to Figure 1.

Upper panel shows the schematic diagram illustrates the design of the dual-color FISH probe targeting *CBX3* (red) and *ALK* (green). The probe spans the fusion breakpoint region to enable detection of *CBX3::ALK* rearrangement.

Lower panel shows validation of the FISH probe in normal cells demonstrating separate biallelic signals for both genes in metaphase spreads and interphase nuclei, confirming the specificity of the probe.

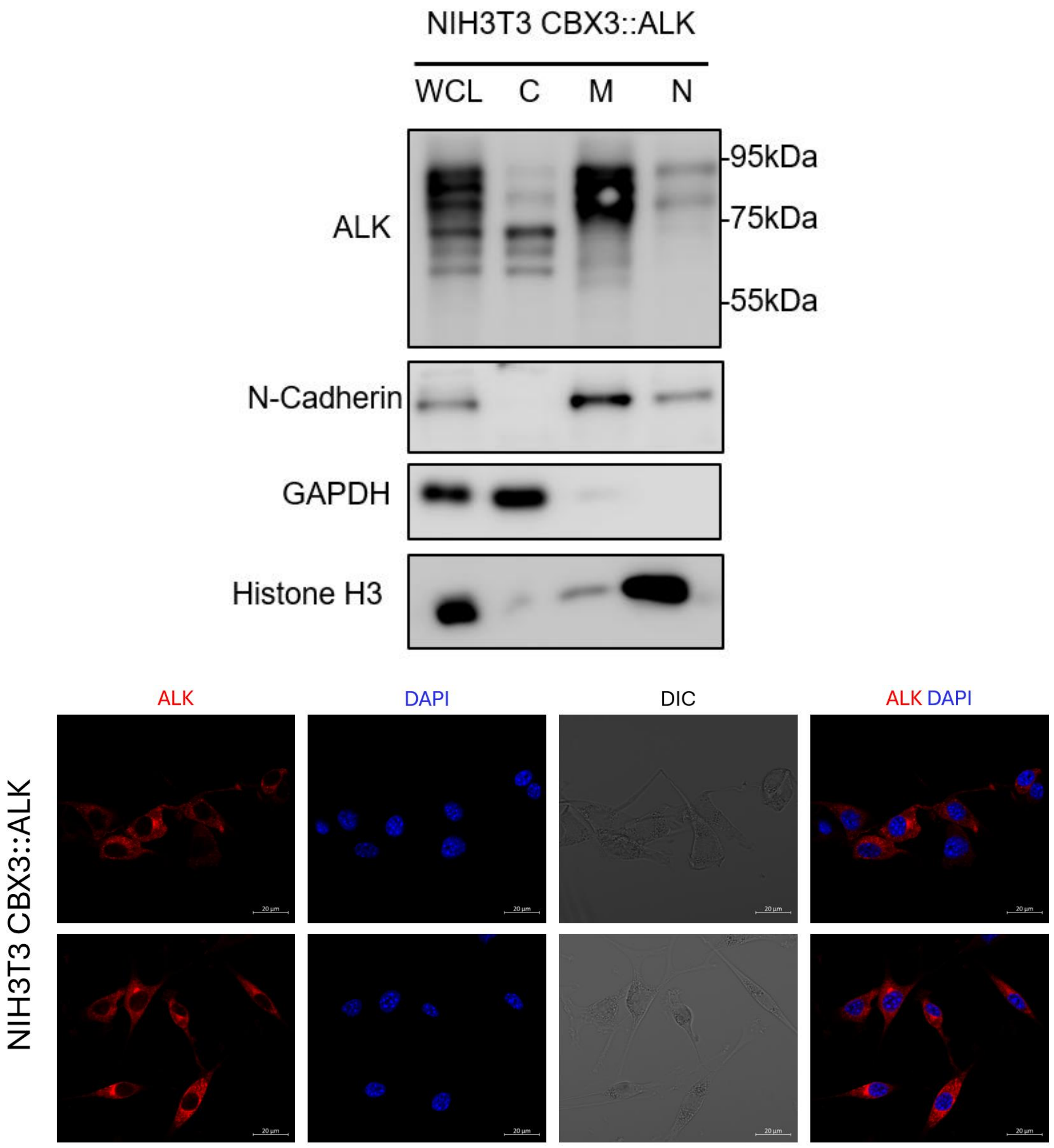

Supplementary Figure S2. Subcellular localization of the ALK isoform generated by the *CBX3::ALK* fusion. Related to Figure 2.

Upper panel shows subcellular fractionation followed by Western blot analysis demonstrating that ALK protein is present in the cytoplasmic and membrane fractions but absent from the nuclear fraction. The detection of ALK protein products with distinct molecular weights across cytoplasmic and membrane compartments suggests the presence of multiple ALK isoforms derived from the *CBX3::ALK* fusion.

Lower panel shows immunofluorescence staining demonstrating that ALK expression derived from the *CBX3::ALK* fusion is restricted to the cytoplasm. Red: FLAG (tagged *CBX3::ALK*); Blue: DAPI (nuclei); DIC: Differential Interference Contrast. Scale bar, 20µm.

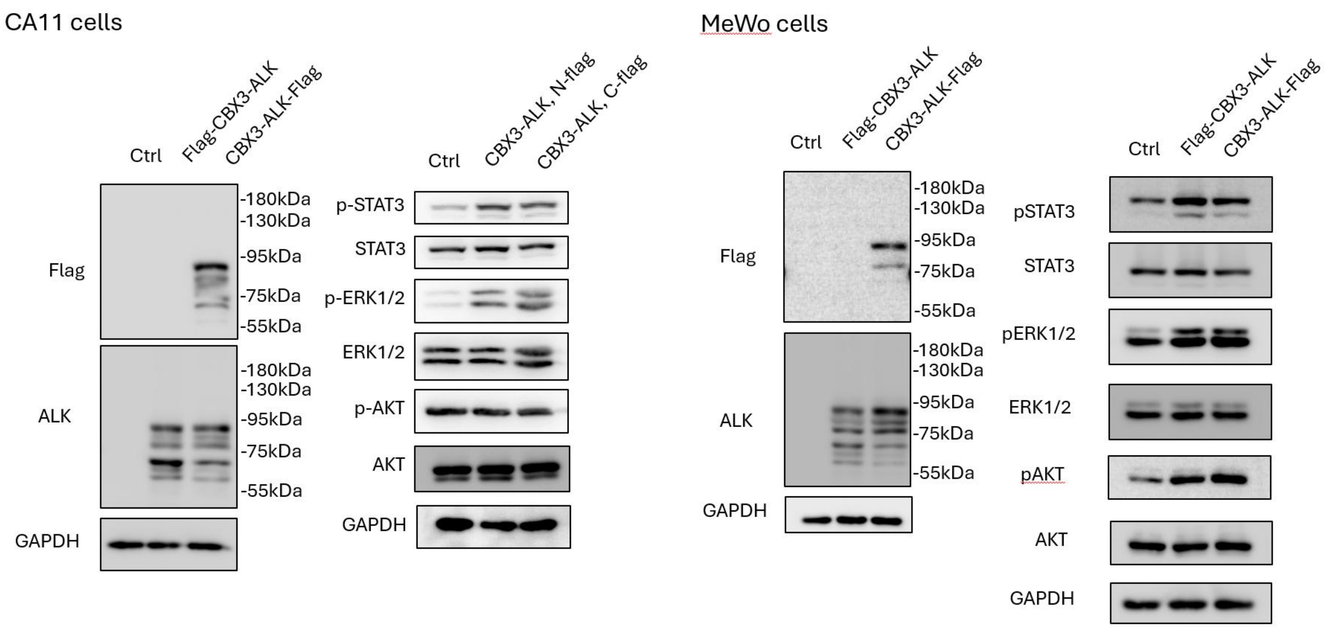

Supplementary Figure S3. ***CBX3::ALK*** **activates downstream signaling in melanoma-relevant human cell lines. Related to Figure 3.**

Western blot analysis of CA11 (acral melanoma; *BRAF/NRAS* wild-type) and MeWo (fibroblast-like metastatic melanoma; *BRAF/NRAS* wild-type) cells stably expressing wild-type *CBX3::ALK*. In both cell models, *CBX3::ALK* expression induced activation of downstream signaling pathways, demonstrated by increased phosphorylation of ALK and key signaling effectors, compared with the empty vector negative control.

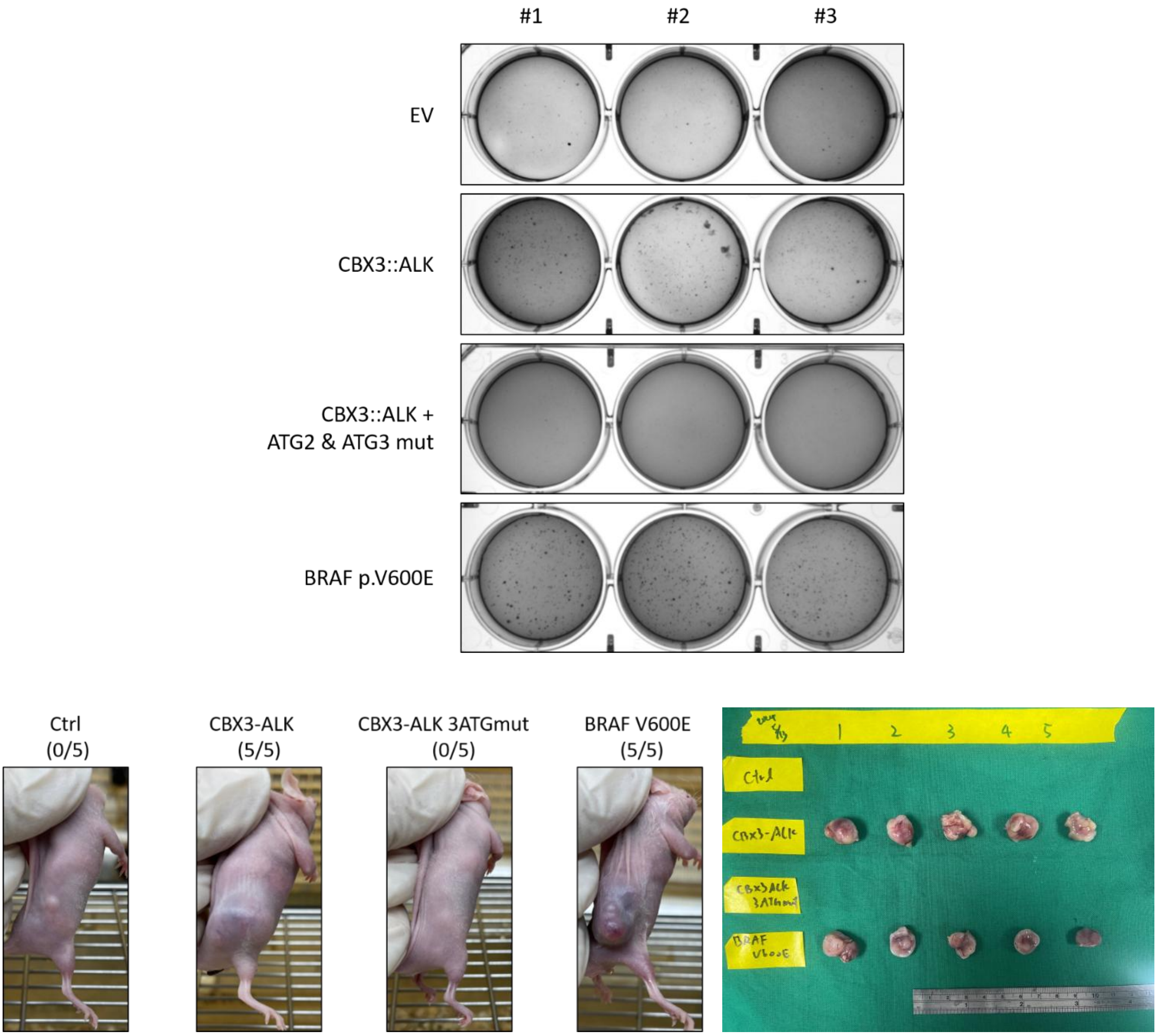

Supplementary Figure S4. ***In vitro*** **and** ***in vivo*** **oncogenic assays of** ***CBX3::ALK*****. Related to Figure 3.**

Upper panel shows soft agar colony formation assay of NIH3T3 cells expressing the indicated constructs. Representative images from three independent experiments are shown at day 14.

Lower panel shows *in vivo* tumorigenicity assay, in which NIH3T3 cells expressing the indicated constructs were subcutaneously inoculated into nude mice to assess tumor growth. Mice were euthanized at day 21, and tumors were harvested for analysis. Experiments were performed with five replicates per group.

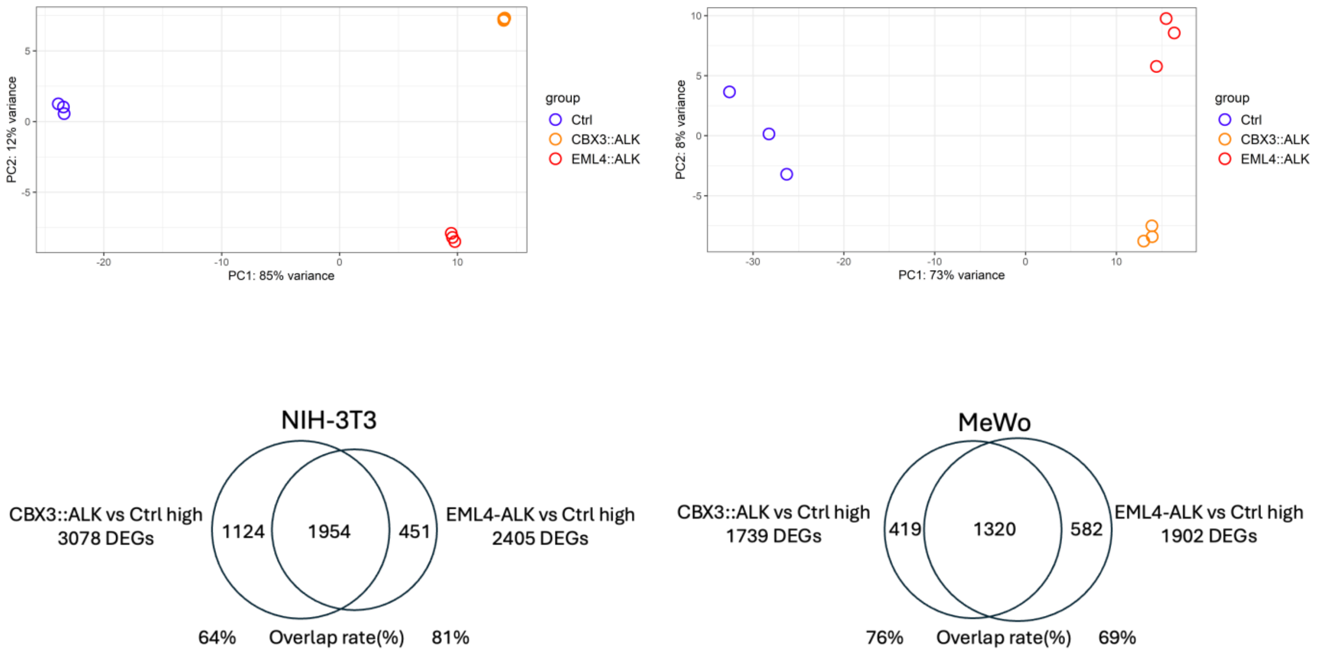

Supplementary Figure S5. Transcriptomic similarities between CBX3::ALK and EML4::ALK. Related to Figure 3. Upper panel shows principal component analysis (PCA) of global gene expression profiles demonstrating clear segregation of samples according to *ALK* fusion status, indicating that *ALK* fusion is a major driver of transcriptomic variation. NIH3T3 cells (left) and MeWo cells (right) are shown.

Lower panel shows Venn diagram illustrating the overlap of significantly up-regulated genes in *CBX3::ALK*- and *EML4::ALK*-expressing cells compared with vector controls, supporting the transcriptomic and functional similarity between the noncanonical out-of-frame *CBX3::ALK* fusion and the canonical in-frame *EML4::ALK* fusion. NIH3T3 cells (left) and MeWo cells (right) are shown.

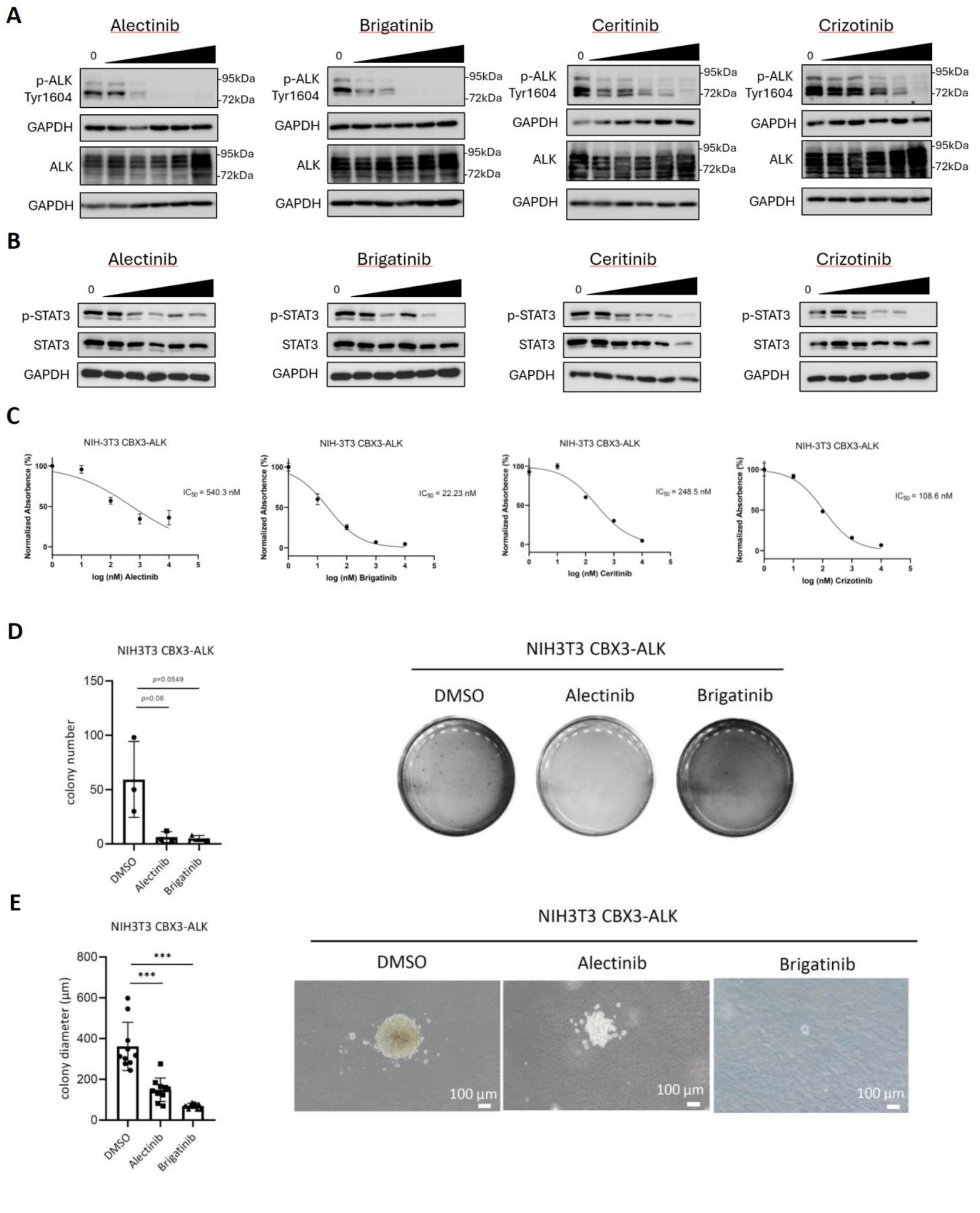

Supplementary Figure S6. ALK inhibitors suppress CBX3::ALK-driven signaling, viability, and transformation. Related to Figure 3.

(A) Western blot analysis of NIH3T3 cells expressing *CBX3::ALK* fusion gene treated with ALK inhibitors (Alectinib, Brigatinib, Ceritinib, and Crizotinib) for 6 hours at increasing concentrations (1, 10, 100, 1,000, and 10,000 nM). Phosphorylated ALK (p-ALK, Tyr1604) and total ALK were analyzed, with GAPDH as the loading control. (B) Western blot analysis of NIH3T3 cells expressing *CBX3::ALK* fusion gene under the same treatment conditions as in panel A.

Phosphorylated STAT3 (p-STAT3, Y705) and total STAT3 were analyzed, with GAPDH as the loading control. (C) Cytotoxicity analysis of ALK inhibitors in *CBX3::ALK*-expressing NIH3T3 cells assessed by CCK-8 assay after 48 hours of treatment. Dose–response curves were fitted using nonlinear regression. Data are presented as mean ± SD (n = 3). (D) Soft agar colony formation assay was performed on *CBX3::ALK* expressing NIH3T3 cells which treated with DMSO control or ALK inhibitors Alectinib 10 μM or Brigatinib 10 μM for 14 days. Cell colonies should be larger than 50 μm diameter. Left, counted colonies number. Right, representative images of soft agar assay in 6-well plate (n=3). The data is presented in mean ± SD, n=3. (Student's t test). (E) Random counted colonies diameter on *CBX3::ALK* expressing NIH3T3 cells which treated with DMSO control or ALK inhibitors Alectinib 10 μM or Brigatinib 10 μM for 14 days. Left, counted colonies diameter. Right, representative images of colonies. The data is presented in mean ± SD, n=10. ***$p < 0.001$ (Student's t test).

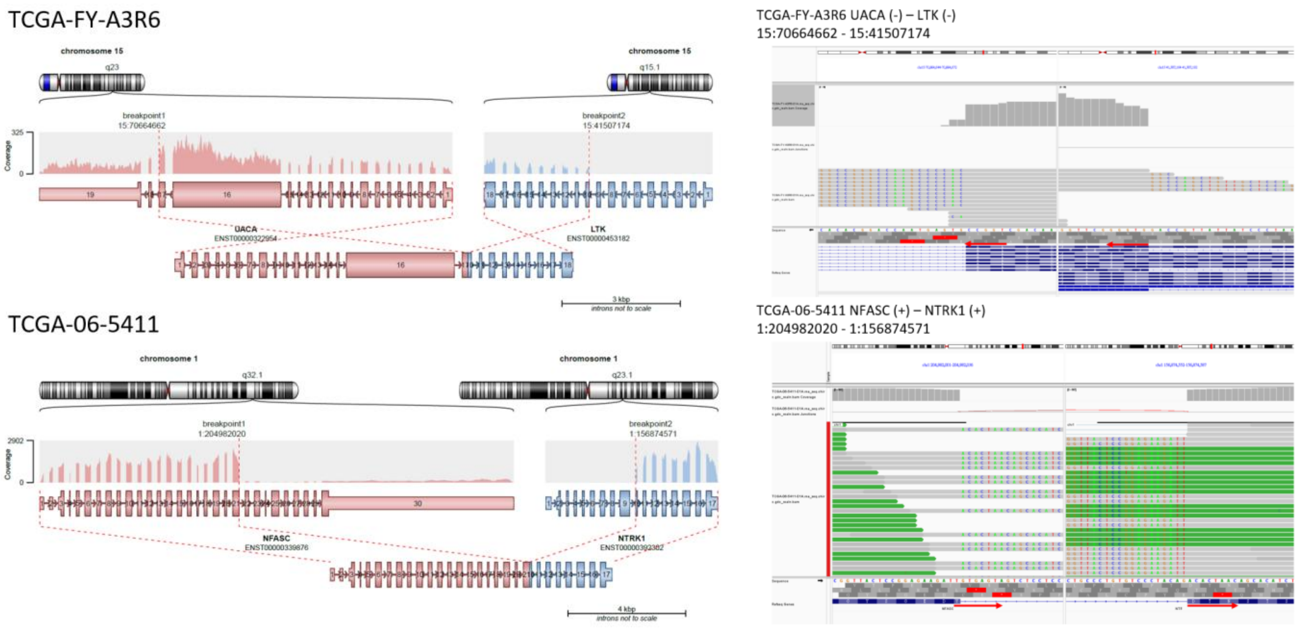

Supplementary Figure S7. Reclassification of previously annotated out-of-frame fusions using RNA-seq and breakpoint analysis. Related to Figure 4.

Left panel: Visualization of fusion gene breakpoints and associated 5′/3′ expression imbalance patterns. De novo analysis of samples previously reported to harbor out-of-frame fusions identified two fusion genes, *UACA::LTK* and *NFASC::NTRK1*, in TCGA-FY-A3R6 (top) and TCGA-06-5411 (bottom), respectively. Fusion breakpoints and expression imbalance were visualized using the Arriba module of the nf-core/rnafusion pipeline (version 3.0.2). The human reference genome (hg38) and gene annotation (Ensembl release 110) were used.

Right panel: In-frame assessment of the same fusion breakpoints using Integrative Genomics Viewer (IGV, version 2.18.0). Manual inspection of fusion junctions demonstrated that both *UACA::LTK* and *NFASC::NTRK1* fusions are in-frame, supporting their reclassification.

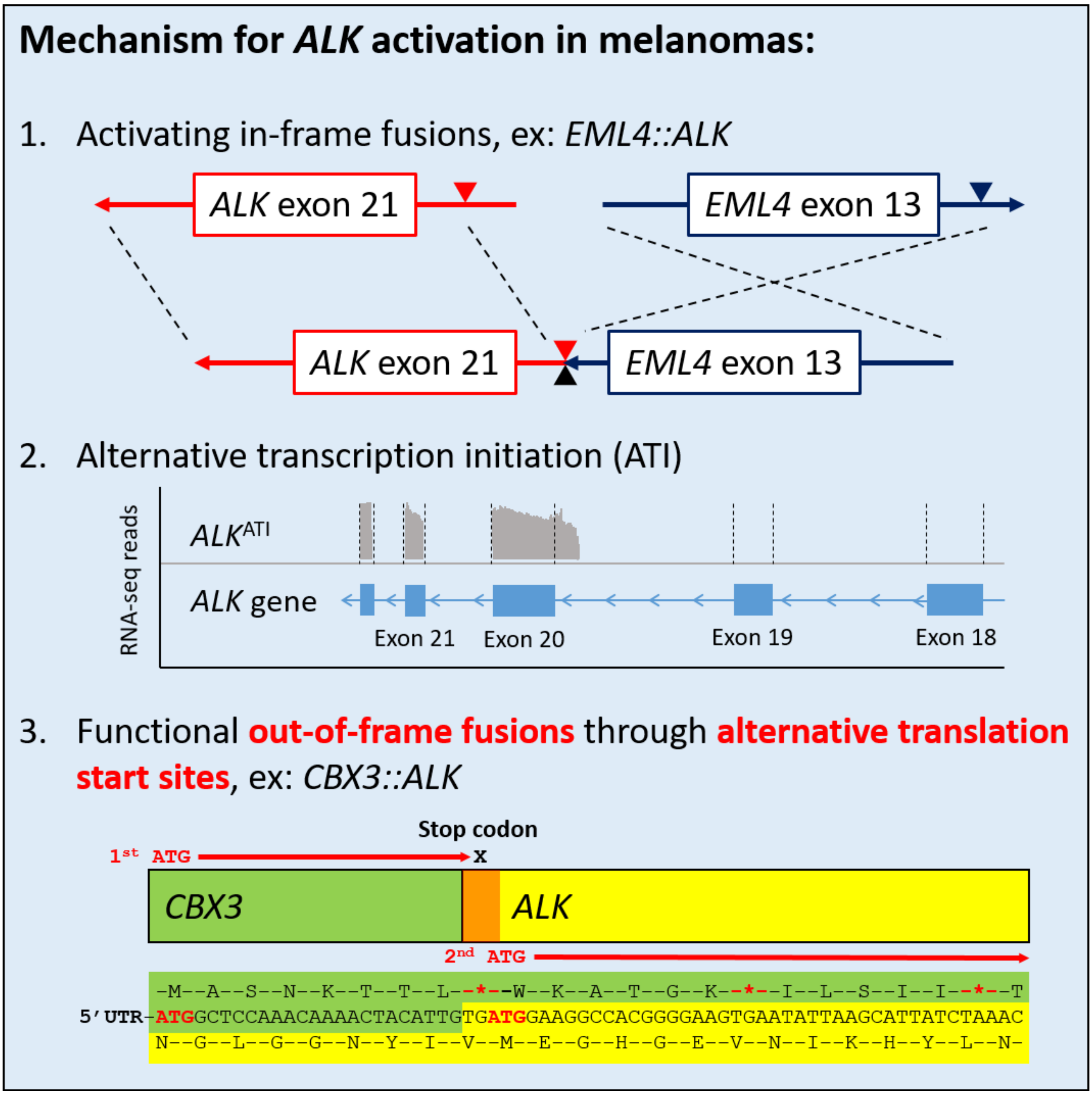

Supplementary Figure S8. Schematic illustration of mechanisms for ALK activation in melanoma. Related to visual abstract.

These include canonical in-frame fusions (e.g., *EML4::ALK*), alternative transcription initiation, and the mechanism described in this study, functional out-of-frame fusions (e.g., *CBX3::ALK*) mediated through alternative translation start sites.

TCTAGAATGGCCTCCAACAAAACTACATTGTGATGGAAGGCCACGGGGAAGTGAATATTAAGCATTATCTAAACTG
CAGTCACTGTGAGGTAGACGAATGTCACATGGACCCTGAAAGCCACAAGGTCATCTGCTTCTGTGACCACGGGAC
GGTGCTGGCTGAGGATGGCGTCTCCTGCATTGTGTCACCCACCCCGGAGCCACACCTGCCACTCTCGCTGATCCTC
TCTGTGGTGACCTCTGCCCTCGTGGCCGCCCTGGTCCTGGCTTTCTCCGGCATCATGATTGTGTACCGCCGGAAGC
ACCAGGAGCTGCAAGCCATGCAGATGGAGCTGCAGAGCCCTGAGTACAAGCTGAGCAAGCTCCGCACCTCGACC
ATCATGACCGACTACAACCCCAACTACTGCTTTGCTGGCAAGACCTCCTCCATCAGTGACCTGAAGGAGGTGCCGC
GGAAAAACATCACCCTCATTCGGGGTCTGGGCCATGGCGCCTTTGGGGAGGTGTATGAAGGCCAGGTGTCCGGA
ATGCCCAACGACCCAAGCCCCCTGCAAGTGGCTGTGAAGACGCTGCCTGAAGTGTGCTCTGAACAGGACGAACT
GGATTTCCTCATGGAAGCCCTGATCATCAGCAAATTCAACCACCAGAACATTGTTCGCTGCATTGGGGTGAGCCTG
CAATCCCTGCCCCGGTTCATCCTGCTGGAGCTCATGGCGGGGGGAGACCTCAAGTCCTTCCTCCGAGAGACCCGC
CCTCGCCCGAGCCAGCCCTCCTCCCTGGCCATGCTGGACCTTCTGCACGTGGCTCGGGACATTGCCTGTGGCTGTC
AGTATTTGGAGGAAAACCACTTCATCCACCGAGACATTGCTGCCAGAAACTGCCTCTTGACCTGTCCAGGCCCTG
GAAGAGTGGCCAAGATTGGAGACTTCGGGATGGCCCGAGACATCTACAGGGCGAGCTACTATAGAAAGGGAGG
CTGTGCCATGCTGCCAGTTAAGTGGATGCCCCCAGAGGCCTTCATGGAAGGAATATTCACTTCTAAAACAGACACA
TGGTCCTTTGGAGTGCTGCTATGGGAAATCTTTTCTCTTGGATATATGCCATACCCCAGCAAAAGCAACCAGGAAG
TTCTGGAGTTTGTCACCAGTGGAGGCCGGATGGACCCACCCAAGAACTGCCCTGGGCCTGTATACCGGATAATGA
CTCAGTGCTGGCAACATCAGCCTGAAGACAGGCCCAACTTTGCCATCATTTTGGAGAGGATTGAATACTGCACCC
AGGACCCGGATGTAATCAACACCGCTTTGCCGATAGAATATGGTCCACTTGTGGAAGAGGAAGAGAAAGTGCCTG
TGAGGCCCAAGGACCCTGAGGGGGTTCCTCCTCTCCTGGTCTCTCAACAGGCAAAACGGGAGGAGGAGCGCAG
CCCAGCTGCCCCACCACCTCTGCCTACCACCTCCTCTGGCAAGGCTGCAAAGAAACCCACAGCTGCAGAGATCTC
TGTTCGAGTCCCTAGAGGGCCGGCCGTGGAAGGGGGACACGTGAATATGGCATTCTCTCAGTCCAACCCTCCTTC
GGAGTTGCACAAGGTCCACGGATCCAGAAACAAGCCCACCAGCTTGTGGAACCCAACGTACGGCTCCTGGTTTA
CAGAGAAACCCACCAAAAAGAATAATCCTATAGCAAAGAAGGAGCCACACGACAGGGGTAACCTGGGGCTGGA
GGGAAGCTGTACTGTCCCACCTAACGTTGCAACTGGGAGACTTCCGGGGGCCTCACTGCTCCTAGAGCCCTCTTC
GCTGACTGCCAATATGAAGGAGGTACCTCTGTTCAGGCTACGTCACTTCCCTTGTGGGAATGTCAATTACGGCTAC
CAGCAACAGGGCTTGCCCTTAGAAGCCGCTACTGCCCCTGGAGCTGGTCATTACGAGGATACCATTCTGAAAAGC
AAGAATAGCATGAACCAGCCTGGGCCCgATTACAAggATgACgACgATAAgTGAGAATTC

Supplementary Figure S9. Sequence of the *CBX3::ALK* C-terminal FLAG-tagged construct. Related to STAR Methods.

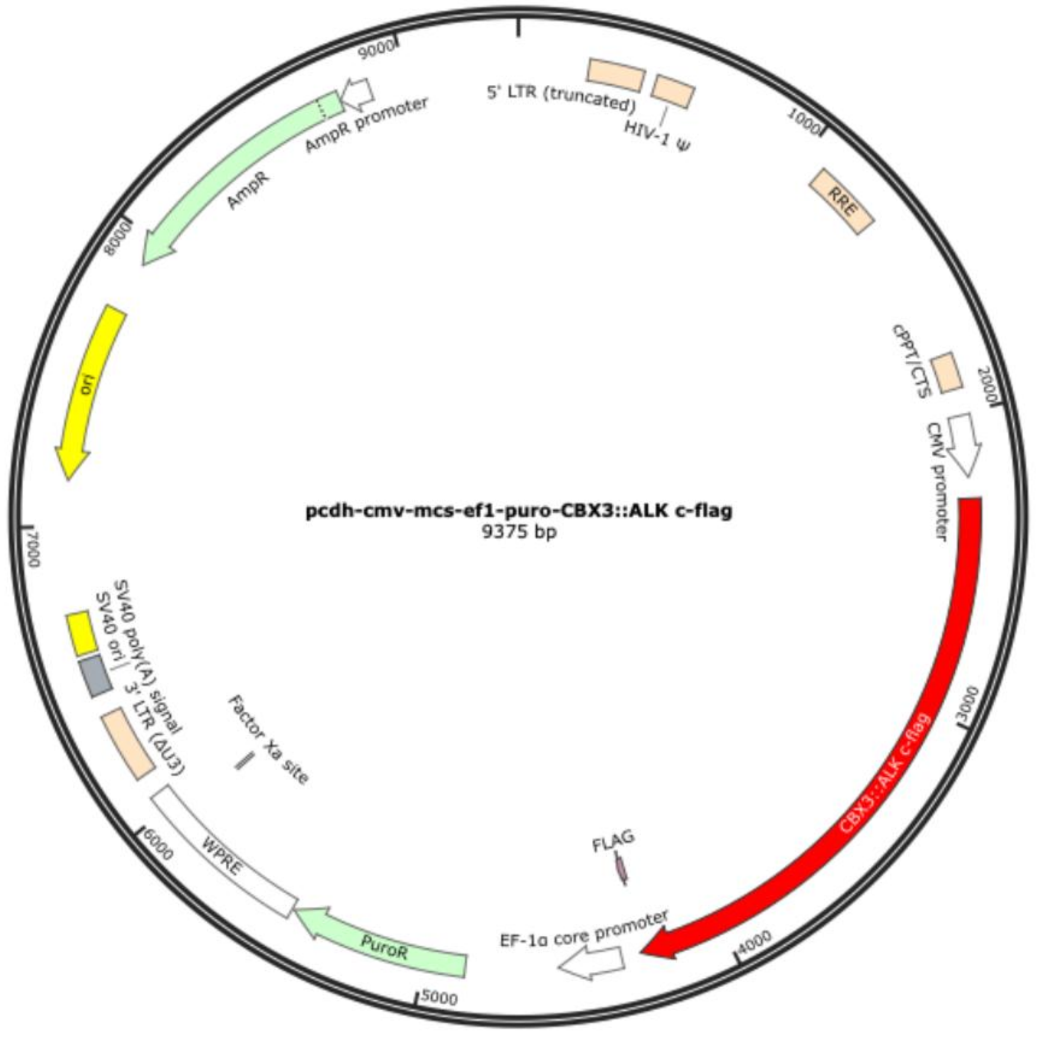

Supplementary Figure S10. Schematic map of the *CBX3::ALK* cDNA expression vector. Related to STAR Methods.

Table S3. Reanalysis of potential out-of-frame gene fusions identified in previous studies. Related to Figure 4.

Fusion[a]	Reference	Sample ID	Strand Orientation[b]	Predicted Reading Frame Effect[c]	Kinase Domain Preservation	5′/3′ Expression Imbalance[d]
RAB3IL1::NRG1	10	TCGA-04-1362	In-strand	Out-of-frame/ In-frame/ In-frame	True	Absent
MDM2::EGFR	10	TCGA-19-262	In-strand	Out-of-frame/ Out-of-frame/ Frameshift	True	Absent
C2orf61::ALK	10	TCGA-23-1027	In-strand	Out-of-frame/ Out-of-frame/ Not detected	True	Absent
UBE2Q2::NRG4	10	TCGA-AC-A3EH	In-strand	Out-of-frame/ Out-of-frame/ Frameshift	False	Absent
HIBADH::BRAF	10	TCGA-BF-A5EP	In-strand	Out-of-frame/ In-frame/ Frameshift	True	Absent
THBS1::NRG1	10	TCGA-CN-5370	In-strand	Out-of-frame/ In-frame/ In-frame	True	Absent
AKAP13::NRG1	10	TCGA-EW-A1P0	In-strand	Out-of-frame/ In-frame/ Frameshift	True	Absent
UACA::LTK	10	TCGA-FY-A3R6	In-strand	Out-of-frame/ In-frame/ In-frame	True	Present
NFASC::NTRK1	11	TCGA-06-5411	In-strand	In-frame/ In-frame/ In-frame	True	Present
NRF1::BRAF	16	MELA_0296	In-strand	In-frame/ In-frame/ Not detected	True	Absent

[a] The 3′ partner genes were selected from the following druggable targets: *AKT2, ALK, AXL, BRAF, CRAF, EGFR, FGFR1, FGFR2, FGFR3, HER2, LTK, MET, NRG1, NTRK1, NTRK2, NTRK3, PDGFB, PDGFRA, PIK3CA, PRKACA, PRKD1, RAF1, RET, ROS1*.

[b] Strand orientation and kinase domain preservation were determined primarily from Arriba outputs.

[c] Predictions for reading frame effects are based on the results of Arriba/FusionCatcher/STAR-Fusion, respectively.

[d] 5′/3′ expression imbalance was manually assessed through visual inspection.

Table S4. RNA sequencing reanalysis. Related to Figure 4.

	p value of Wilcoxon rank-sum test		
	**5′ Partner Gene**	**3′ Partner Gene**	**5′/3′ Expression Imbalance**
TCGA-04-1362_RAB3IL1-NRG1	0.2667	0.0095	Absent
TCGA-19-2624_MDM2-EGFR	0.1497	0.0000	Absent
TCGA-23-1027_STPG4-ALK	0.5000	0.0551	Absent
TCGA-AC-A3EH_UBE2Q2-NRG4	1.0000	NA	Absent
TCGA-BF-A5EP_HIBADH-BRAF	0.2000	0.0306	Absent
TCGA-CN-5370_THBS1-NRG1	0.9699	0.7922	Absent
TCGA-DJ-A4UT_MALAT1-ALK	1.0000	0.0000	Absent
TCGA-EW-A1P0_AKAP13-NRG1	0.2679	0.1818	Absent
TCGA-FY-A3R6_UACA-LTK	0.0147	0.0058	Present
TCGA-06-5411_NFASC-NTRK1	0.0000	0.0006	Present
MELA_0296_NRF1-BRAF	0.1255	0.2861	Absent
